# Dynamic Communications Between GABA_A_ Switch, Local Connectivity, and Synapses During Cortical Development: A Computational Study

**DOI:** 10.3389/fncel.2018.00468

**Published:** 2018-12-17

**Authors:** Radwa Khalil, Ahmed A. Karim, Eman Khedr, Marie Moftah, Ahmed A. Moustafa

**Affiliations:** ^1^Department of Psychology and Methods, Jacobs University Bremen, Bremen, Germany; ^2^University Clinic of Psychiatry and Psychotherapy, Tübingen, Germany; ^3^Department of Neuropsychiatry, Faculty of Medicine, Assiut University, Assiut, Egypt; ^4^Zoology Department, Faculty of Science, Alexandria University, Alexandria, Egypt; ^5^MARCS Institute for Brain and Behaviour, Western Sydney University, Sydney, NSW, Australia; ^6^Department of Social Sciences, College of Arts and Sciences, Qatar University, Doha, Qatar

**Keywords:** firing rate activity, local connectivity, *in vitro*, dynamical synapses, GABAA signaling, cortical development, STD, STF

## Abstract

Several factors regulate cortical development, such as changes in local connectivity and the influences of dynamical synapses. In this study, we simulated various factors affecting the regulation of neural network activity during cortical development. Previous studies have shown that during early cortical development, the reversal potential of GABA_A_ shifts from depolarizing to hyperpolarizing. Here we provide the first integrative computational model to simulate the combined effects of these factors in a unified framework (building on our prior work: Khalil et al., 2017a,b). In the current study, we extend our model to monitor firing activity in response to the excitatory action of GABA_A_. Precisely, we created a Spiking Neural Network model that included certain biophysical parameters for lateral connectivity (distance between adjacent neurons) and nearby local connectivity (complex connections involving those between neuronal groups). We simulated different network scenarios (for immature and mature conditions) based on these biophysical parameters. Then, we implemented two forms of Short-term synaptic plasticity (depression and facilitation). Each form has two distinct kinds according to its synaptic time constant value. Finally, in both sets of networks, we compared firing rate activity responses before and after simulating dynamical synapses. Based on simulation results, we found that the modulation effect of dynamical synapses for evaluating and shaping the firing activity of the neural network is strongly dependent on the physiological state of GABA_A_. Moreover, the STP mechanism acts differently in every network scenario, mirroring the crucial modulating roles of these critical parameters during cortical development. Clinical implications for pathological alterations of GABAergic signaling in neurological and psychiatric disorders are discussed.

## Introduction

One of the most remarkable discoveries in the developing brain is the shift of actions conducted by the neurotransmitter GABA that inhibits adult neurons but excites immature ones due to an initially higher intercellular chloride concentration [Cl^−^]_i_, leading to depolarizing and excitatory actions of GABA instead of hyperpolarizing and inhibitory actions (Ben-Ari, 2002, 2007a; Ben-Ari et al., 2007b, 2012). Thus, the development of the GABAergic system is vital for the harmony between excitatory and inhibitory neurons in adult cortical systems (Dichter, 1980; Buzsáki and Draguhn, 2004; Kato-Negishi et al., 2004; Ben-Ari, 2007a; Ben-Ari et al., 2007b). Experimental evidence revealed that the acute activity-dependent modulation of the neuron-specific potassium-chloride cotransporter (KCC2) might provide a central mechanism for a partial reversal of the excitation-to-inhibition change of GABAergic transmission (Ganguly et al., 2001; Ben-Ari, 2002; Fiumelli et al., 2005). However, transcriptional regulation of KCC2 expression and post-translational modification of KCC2 function might have a differential augmentation to the plasticity of the GABAergic system (Fiumelli et al., 2005). The low expression of KCC2 during early development is due to the rise of post-synaptic intracellular chloride [Cl–]^i^ in young neurons; thus, GABAergic transmission is depolarizing and excitatory (Ben-Ari, 2002). Later during development, the up-regulation of KCC2 [Cl–]^i^ generates a shift in Cl-equilibrium potential (E_Cl_) toward more negative levels, switching GABAergic transmission from excitatory to inhibitory (Ben-Ari, 2002). Therefore, the depolarizing action of GABA itself promotes the developmental up-regulation of KCC2 through Ca^2+^-dependent transcriptional regulation (Ganguly et al., 2001).

It has been argued that synaptogenesis coincides reasonably well with the initiation of dendritic development and that the density of synapses significantly rises at least until the end of the third week *in vitro* (Ito et al., 2010). This rise in the synaptic density occurs despite the decline in neural density (Ito et al., 2010). Consequently, lateral connectivity (distance between adjacent neurons) might lead to extensive modification of the nearby local connectivity (complex connections involving those between neuronal groups) leading to the enhancement and fine-tuning neural activity (Bienenstock, 1996; Sporns et al., 2000).

Short-term synaptic plasticity (STP) plays a crucial role in sustaining the neural network activity through inducing changes in synaptic efficacy over time. This maintenance is a consequence of modulating the timing of signal processing through mediating the driven Poisson input frequency [IF (Hz)] and filtering signal propagation (Tsodyks and Markram, 1997; Tsodyks et al., 1998; Loebel and Tsodyks, 2002). There are two forms of STP: Short-term depression (STD) and Short-term facilitation (STF).

Various findings shed light on the impact of the external input frequency [IF (Hz)] on the regulation of several developmental processes. Buzsáki and Draguhn (2004) pointed that network oscillations bias input selection because they transiently assort neurons into assemblies. This input selectivity enhances the synaptic plasticity of these neurons and co-operatively maintain their temporal signaling processes of information (Buzsáki and Draguhn, 2004).

Despite the crucial influence of these parameters, there is a lack of adequate experimental evidence in addressing the correlations between them. Hence, it is necessary to afford a dynamical network model as a predictive tool to evaluate and understand the dynamical interactions of these parameters. This modeling tool would highlight our current understanding of monitoring the neural firing rate activity (Hz) through the physiological development of the GABA_A_ reversal potential. Therefore, we propose an *in silico* model of the effects of immature and mature GABA_A_ signaling.

Based on our previous work on dynamical synapses (Khalil et al., 2017a,b) and to better explain the effects of dynamical synapses on cortical network development, we extended our modeling study to observe firing activity in response to the excitatory action of GABA_A_. Accordingly, we considered the reversal potential of GABA_A_ to be excitatory in the immature condition and inhibitory in the mature condition (Ben-Ari, 2002, 2007a; Ben-Ari et al., 2007b, 2012).

We targeted the impact of GABA_A_ signaling before and after the physiological maturation crossing the dynamical switch from excitation to inhibition (Dichter, 1980; Buzsáki and Draguhn, 2004; Kato-Negishi et al., 2004; Ben-Ari, 2007a; Ben-Ari et al., 2007b). Here, we divided our model into a number of network scenarios by introducing certain proportions of lateral connectivity (distance between adjacent neurons) and the nearby local connectivity (complex connections involving those between neuronal groups). Moreover, we introduced distinct values of Poisson input frequency [IF (Hz); varying from 5 to 100 Hz, with 5 Hz interval] per network scenario. We then conducted the simulation for each network scenario for immature and mature conditions. We performed each simulation run before and after implementing STP. Therefore, we measured the effects of dynamical synapses on modulating the produced firing activity.

## Methods

**Figure 1:**
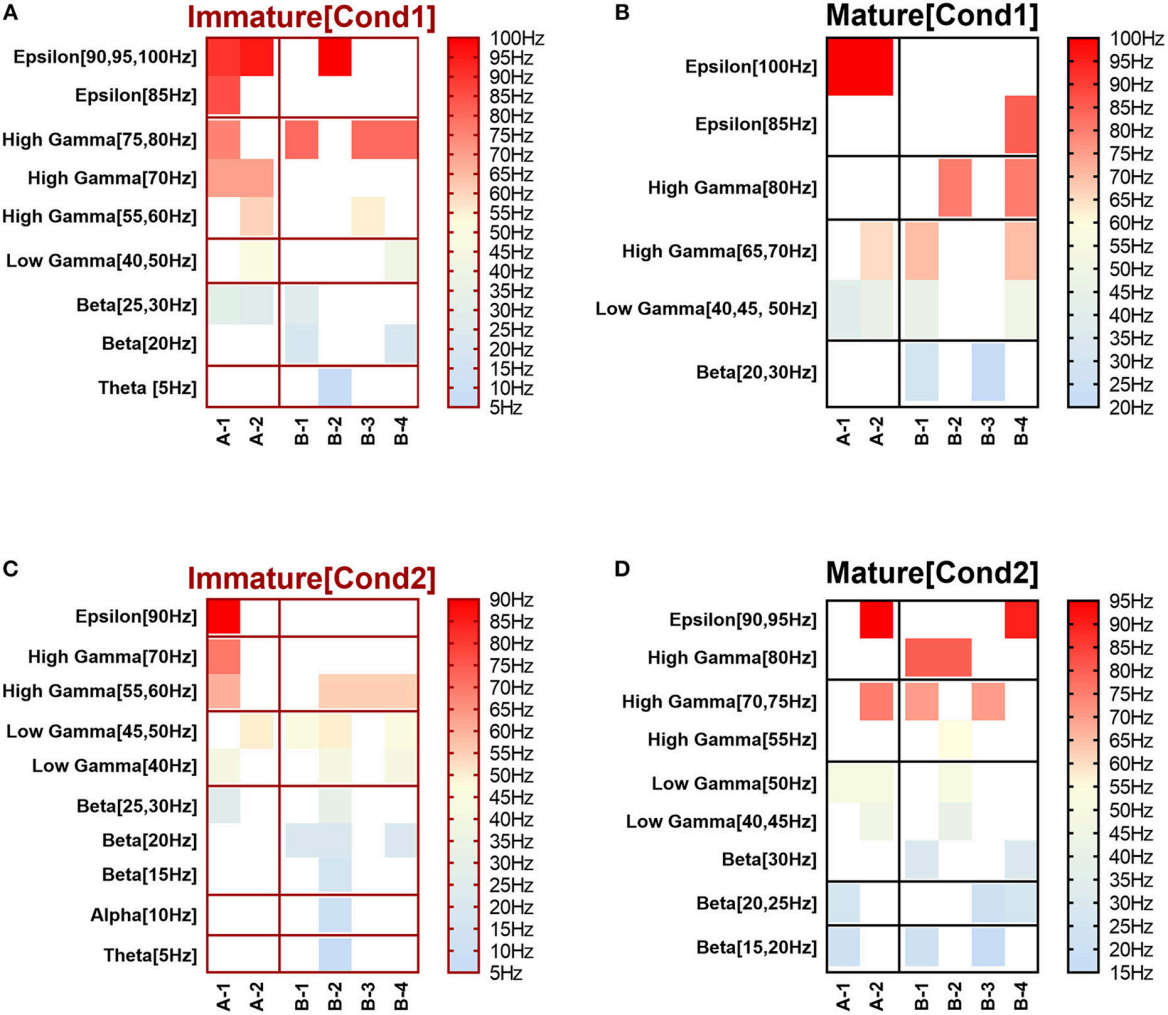
Selected input frequencies [IF (Hz)] that succeeded across all network scenarios of both conditions: immature **(A,C)** and mature networks **(B,D)**, in triggering modulation effect after implementing depressing and facilitating dynamical synapses. Ranges are theta [3–7 Hz], alpha [7–12 Hz], beta [12–30 Hz], gamma (low [30–50 Hz] and high [50–80 Hz]), and epsilon [80–250 Hz] (throughout Figure 3A of Ji et al., 2013). **(A,B)** refer to condition 1, which indicates the predicted modulated responses (in response to STP) while **(C,D)** relate to condition 2 (unpredicted modulated responses in response to STP).

Here, we designed a Spike Neural Network (SNN) model to monitor the neural firing activity responses within the two physiological states of GABA_A_. Following fine-tuning and optimization of our model (see Supplementary Materials: Figures S.1.1, S.1.2 in Khalil et al., 2017a,b), we systematically segregated it into several scenarios (Figure 1; Appendix A, C.2: Network Scenario). For each network scenario, we relied on biophysical parameters that had been intensively used in various experiments and biophysical studies (Appendix A, E: Model Parameters, see also Khalil et al., 2017a,b). We then implemented two types of STP, with fluctuations in the membrane time constant. Consequently, we performed the simulation with and without STP (Appendix A, F: Short Term Synaptic Plasticity).

We performed a three-way ANOVA to examine the effects of network scenarios, maturation of the network (i.e., immature and mature based on the reversal potential state of GABA_A_) and the STP class (i.e., STD1, STD2, STF1, and STF2) for each IF value (i.e., 5, 10, …, 100 Hz ) on the firing rate activity (Hz). There were significant three-way interactions. Subsequently, we performed a two-way ANOVA as a follow up statistical test. Also, we segregated the firing rate activity (Hz) according to the observed modulated response toward both classes of dynamical synapses into the predicted and unpredicted one based on previous STP studies (Tsodyks and Markram, 1997; Tsodyks et al., 1998; Loebel and Tsodyks, 2002). Finally, we measured the significant level of the produced firing activity (Hz) before and after implementing dynamical synapses (i.e., after dynamically varying the synaptic status).

### Network Description

#### Neuron Model

The neural network is composed of *N* = 3,000 neurons [2,400 excitatory (n_exc) and 600 inhibitory (n_inh)]. These neurons are simulated using leaky integrate and fire equations, which have been used in previous studies (Brette and Gerstner, 2005; Plesser and Diesmann, 2009; Ahmed et al., 2014; Grüning and Bohte, 2014; Abbott et al., 2016). The involved biophysical parameters have been previously reported (Appendix A, E: Model Parameters). To ensure the robustness of our model and to verify that data were not subjectively biased we changed the refractory period value (τ_ref_) in further simulations. We also performed additional simulations with up to 12,000 neurons in the initial trial stages to examine the legitimacy of our simulations.

#### Synapse Model

We modeled synaptic interactions between neurons (*N*) as transient conductance changes, in which we considered the synaptic time course to increase instantaneously accompanied by an exponential decay. We selectively chose the following synaptic time constant values: τ_exc_ = 5 ms and τ_inh_ = 10 ms for glutamatergic excitation (AMPA) and GABAergic inhibition (GABA_A_), respectively. As for AMPA synapses, we implemented it due to its critical role in regulating neural network activity during development (Bredt and Nicholl, 2003; Hall and Ghosh, 2008; Kessels and Malinow, 2009; Santos et al., 2009; Czöndör and Thoumine, 2013; Hanse et al., 2013). Next, we set reversal potentials to E_exc_ = 0 mV, whilst E_GABA_ = −70 mV (inhibitory GABA_A_) and −40 mV (excitatory GABA_A_). We used 4 nS for excitatory conductance and a balance of g_inh_ = 64 nS for inhibitory conductance unless stated otherwise. Lastly, we used 200 nS conductance for external Poisson input (g_ext_) (Appendix A, E: Model Parameters).

#### Poisson Input Frequency (Hz)

Initially, we examined the responses of the network model with 1 to 10 Hz values of Poisson input frequency (IF) with an interval of 1 Hz. Since this interval did not provoke considerable variations in the firing responses, we decided to expand it to 5 to 100 Hz with a 5 Hz interval (Appendix A, D: Input and E: Model Parameters).

#### Connectivity Profile

Similar to the model of Yger et al. (2011), we considered neurons of our SNN model as being connected with a distance-dependent probability following a Gaussian profile (Yger et al., 2011). Nevertheless, it is acknowledged that actual connectivity between neurons is less isotropic and homogeneous (i.e., the orientation maps and the patchy horizontal connectivity in V1: Gilbert and Wiesel, 1983). Thus, the Gaussian profile is a beneficial description for a small cortical area when long-range interactions are disregarded (Yger et al., 2011). Accordingly, each neuron communicates with the remaining neurons of the network with a “2D Gaussian probability function” while we applied periodic boundary conditions during the simulation to withdraw any boundary consequences (Yger et al., 2011).

To test the efficiency of connectivity between excitatory and inhibitory populations, we examined the network response at mV level when GABA_A_ reversal potential is inhibitory (Supplementary Materials in Khalil et al., 2017b). For the sake of picking the most optimal profile of connectivity between inhibitory and excitatory neuronal populations, we focused on outlining the connectivity structure of our network before implementing STP (Presentation 1). Accordingly, we applied a systematic procedure, in which we measured the sub-threshold voltage of neurons in response to variations in the connectivity profile between inhibitory and excitatory neuronal population (Figure S.1 in Khalil et al., 2017b). Then, we monitored the firing activity (Hz) for selecting the “best-fit” condition for the connectivity profile at an mV level (Figure S.2 of Khalil et al., 2017b). For structuring the connectivity profile, we used Gaussian-distributed local connectivity according to the probabilistic and topological features of our SNN model. We utilized this strategy for each state of GABA_A_ (i.e., depolarization and hyperpolarization). Here, we assumed that the structure of the connectivity matrix remains fixed following the network initialization. We relied on one set of connection with comparable properties. It is presented in two values, a local density of dendritic arborization (ϵ) (i.e., lateral connectivity (distance between adjacent neurons) between neurons) and a lateral spread length between adjacent neurons (σ_c_) [i.e., the nearby local connectivity (complex connections involving those between neuronal groups)]. Similar to the topological neural model of Yger et al. (2011), we systematically used two main parameters: the spatial extent of the Gaussian profile for the recurrent connections σ_c_ and the local density of dendritic arborization while we kept the balance between excitatory and inhibitory synaptic strength. In order to gain further insight into the performance of the network structure, we primary utilized ranges of percentages for local density of dendritic arborization {1, 2, …, 21%}, which point to the average number of synapses per neuron and their strength. This has been shown to be one of the principal relevant parameters for macroscopic quantities (Yger et al., 2011). Finally, we used proportions for a lateral spread length between neighbor neurons {1, 2, …,10%} and we selected reciprocal values for both parameters, which resulted in eliciting a reasonable firing activity response (Figure 1 in Khalil et al., 2017a,b, see also Appendix A, C: Connectivity). The design of the network scenarios relies on the variations in the percentages of (ϵ) and (σ_c_). (ϵ) and (σ_c_) refers to the percentages of the local density of dendritic arborization (i.e., lateral connectivity between neurons) and the percentages of lateral spread length between neighbor neurons (i.e., the nearby local connectivity), respectively. Both network scenarios; (A-1) and (A-2) have the same proportion of ϵ (1%), but two distinct σ_c_ percentages (9%) and (10%), respectively. On the other hand, (B-1), (B-2), (B-3), and (B-4) have the same σ_c_ proportion (1%), but four different ϵ percentages (9%), (10%), (19%), and (20%), respectively (see C.2: Network Scenarios, see also Figure 1 of Khalil et al., 2017b).

#### Short-Term Synaptic Plasticity (STP)

We adopted the typical Integrate-and-Fire balanced network configuration comprising a 4:1 ratio between excitatory and inhibitory neurons (Brunel, 2000; Nordlie et al., 2009; Vogels et al., 2011; Yger et al., 2011; Kriener et al., 2013).

We applied STP to our neural network model through implementing STD and STF. For each type, we studied two further kinds of STP based on their differential time constant of synaptic refractoriness, namely STD1, STD2, STF1, and STF2 for depressing and facilitating synapses, respectively (Appendix A, F: Short Term Synaptic Plasticity). For both forms of depressing synapses, we set the synaptic time constant for depression to 100 ms while we changed it for facilitation to 1 ms for STD1 and 10 ms for STD2. Likewise, we systematically used the same values for facilitating synapses, in which we set the time constant for facilitating synapses to 100 ms and changed it for depression to 1 ms for STF1 and 10 ms for STF2.

### Algorithms and Interpretation of Model Biophysical Parameters

#### Conductance-Based Leaky Integrate and Fire Algorithm (LIF)

We used SNN model (Vreeken, 2002; Yger et al., 2011; Stimberg et al., 2014) with LIF neurons and balanced excitatory and inhibitory connections (80% excitation, 20% inhibition). Our neural model consisted of 3,000 IIF neurons, characterized by a membrane time constant, τ_m_ = 20 ms, and resting membrane potential, V_rest_ = −74 mV. For more details about this model, see Destexhe (1997).

(1.1a) $$\begin{array}{l} \frac{dV(t)}{dt}=(El-V(t)/\tau_{m}+(g_{exc}(t)(Ee-V\left(t\right))\\ \;+g{\;}_{inh}(t)(E_{GABA}-V(t))/c\_m\;\} \end{array}$$

(1.1b) $$\begin{array}{l} \;\frac{dV\left(t\right)}{dt}=(El-V(t)/\tau_{m}+(g{\;}_{exc}(t)(Ee-V\left(t\right))\\ \;+g_{inh}(t)(Ei-V(t))/c\_m\} \end{array}$$

(1.2) $$\frac{d{\text{g}}_{exc}}{dt}=-g_{exc}/\tau_{exc}\text{ and}\;\frac{d{\text{g}}_{inh}}{dt}=-g_{inh}\;/\tau_{inh}\}$$

The membrane potential of LIF neuron was determined by Equations (1.1a) (for immature network condition), (1.1b) (for mature network condition) and (1.2) (for synaptic conductance), see also Appendix A for more model description and model parameters. Equation (1.1a); El,τ_*m*_, *g*_*exc*_, *Ee*, _*inh*_, *E*_*GABA*_ and *c*_*m*_ refer to leak reversal potential (EI = −70.6 mV), membrane time constant (τ_m_ = 20 ms), decay constant of AMPA-type conductance (g_exc_ = 4 nS), excitatory reversal potential for AMPA (Ee = 0 mV), decay constant of GABA-type conductance (g_inh_ = 64nS), GABA_A_′ reversal potential for immature neocortical network (E_GABA_ = −40 mV), respectively. Equation (1.1b) is similar to Equation (1.1a), but Ei is instead of E_GABA_ (i.e., GABA_A_′ reversal potential for the mature neocortical network [Ei = −70 mV)]. Through Equation (1.2); g_exc_, τ_exc_, g_inh_, τ_inh_ refer to decay constant of AMPA-type conductance (excitatory glutamatergic conductance (AMPA); g_exc_ = 4 nS), glutamatergic synaptic time constant for AMPA (τ_exc_ = 5 ms), decay constant of GABA-type conductance (inhibitory GABAergic conductance (GABA_A_); g_inh_ = 64 nS) and GABAergic synaptic time constant for GABA_A_(τ_inh_ = 10 ms), respectively. When the membrane potential reaches a threshold value (V_t_) of −50.4 mV, the neuron fires and the resting membrane potential(V_rest_) remains at −74 mV for a refractory period (τ_ref_) of 5 ms, see also A: Model Summary and E: Model Parameters.

#### Gaussian-Distributed Local Connectivity Profile

Similar to the connectivity profile of Yger et al. (2011), each neuron sparsely communicates with the rest of the neurons through a connection probability. This probability depends on the distance *r*_*ij*_ between two neurons according to the Gaussian profile (Yger et al., 2011).

(2.1) $$\;p_{ij}=\;e^{-\frac{r_{ij}^{2}}{2\delta_{c}^{2}}\;}\}$$

${\text{σ}}_{\text{c}}^{\text{2}}$ represents the variance of the connectivity profile, which refers to the spatial spread of the Gaussian profile (Yger et al., 2011). For each neuron, the incoming connections (see also Equation 1.4, A: Model Summary, Synapse Model and Synaptic Dynamics) were created by randomly selecting other neurons in the network based on the probability of developing a projection to other neurons according to a rejection method based on the Gaussian profile (Yger et al., 2011). σ_c_ refers to the spatial extent of the Gaussian profile for recurrent connections (i.e., local connectivity). The neuron density in the network is uniform and connections are restricted to the maximal value L/$\sqrt{\text{2}}$, the probability of finding one neuron at distance *r* equals:

$$\begin{array}{l} \;P(r)=\frac{2\pi r}{L^{2}}\;Ifr\leq \frac{L}{2}\\ P(r)=\frac{r(2\pi -8arccos(\frac{L}{2r})}{L^{2}}\;If\frac{L}{2}\;<r\leq \frac{L}{\sqrt{2}}\\ \;P(r)=0\;If\frac{L}{\sqrt{2}}<r \end{array}$$

The quantity of the established connections at r distance and the distance-dependent connection probability likelihood, given by the Gaussian profile, are thus:

(2.2) $$\;N_{realized}\left(r\right)=NP\left(r\right)exp(-r^{2}/2\sigma_{c}^{2})\}$$

With the normalization condition$\overset{L/\sqrt{}2}{\underset{0}{\int }}N_{realized}\;\left(r\right)dr=\in N$. The probability of connection is therefore given by Equation (2.3):

(2.3) $$\;\rho \left(r\right)\;=P\left(r\right)exp\left(-\frac{r^{2}}{2\sigma_{c}^{2}}\right)\}$$

Here, $\overset{L/\sqrt{}2}{\underset{0}{\int }}\text{ρ}\left(r\right)dr=\in .$

The distributions of **ρ*(r)***are persistently influenced by **σ**_***c***_. Indeed, these functions relate to the Gaussian profile and the likelihood ***P(r)***of finding a pair of neurons for a given distance following a standardizing condition (Yger et al., 2011). The total number of external synapses received by each neuron represented by “***K***,” which relates to the number of recurrent synapses (Yger et al., 2011). We fixed K per neuron, and therefore whatever the σ_c_ value is, each neuron kept an equal number of incoming synapses (Yger et al., 2011; see also Equations 1.3, 1.4; A: Model Summary, Synapse Model and Synaptic Dynamics). The variable ϵ*_* is defined as the local density of dendritic arborization. We considered neurite density (i.e., the local density of dendritic arborization) in the network, confining connections to the maximal value $\frac{\text{L}}{\sqrt{\text{2}}}$ with the likelihood of finding one neuron at r distance as mentioned above.

#### Non-homogenous Propagation Delay

We used non-homogeneous delays, which depended linearly on the distances r_ij_ through

(2.4) $$\;d_{ij}=d_{syn\;}+\frac{rij}{v}\}$$

A value of 0.1–0.5 m/s for v is usually reported (Bringuier et al., 1999; González-Burgos et al., 2000), and in all simulations, we used a propagation speed (v) = 0.5 m/s, and d_syn_ = 0.2 ms.

Anatomical and physiological studies (Bringuier et al., 1999; González-Burgos et al., 2000) have reported standardized values of 0.1–0.5 m/s for conduction delays. Also, similar values can be recorded in voltage-sensitive dye imaging, where activity waves propagate at a comparable speed (Grinvald et al., 1994; Benucci et al., 2007). Patch recordings *in vitro* corroborate the fact that this delay linearly scales, as a function of distance (Larkum et al., 2001) when considering the propagation from dendrites to soma. Thus, even for a small patch of cortex of 1 mm^2^, with a synaptic delay (d_syn_) of 0.2 ms (due to neurotransmitter release), conduction delays are widely distributed and should not be neglected. We thus built our network as an artificial square lattice of 1 mm^2^, and we picked a propagation speed of v = 0.5 m/s.

### Model Implementation and Simulation

We performed all simulations using Brian v1.4.1 (Goodman and Brette, 2008, 2009) and the PyNN interface (Davison et al., 2009). We measured the neural firing rate activity (Hz) and discarded the first 50 s of recording from the analysis to avoid potential onset transients, as suggested by Nawrot et al. (2007). Then, we imported all the simulated firing rate values to Graph Pad Prism-software (Graph Pad Prism version 7.00 for Windows, Graph Pad Software, La Jolla California USA, www.graphpad.com). We incorporated 12 simulation trials for each step. All analyses were conducted using Graph Pad Prism-software and SPSS.

### Statistical Analysis

#### Measures of the Modulated Responses Through two Physiological States of GABA_A_

In order to investigate the effects of three crucial factors on the firing rate activity (Hz), we performed a 3-way ANOVA. These factors refer to network scenarios, maturation of the network (i.e., immature and mature) and STP for each value of IF (i.e., 5, 10, …, 100 Hz.). This 3-factor-ANOVA revealed significant 3-way interactions, which are shown in Tables 1–4 and summarized as follows. (1) 5 and 10 Hz IF (theta and alpha range) induced a significant effect (*P* < 0.0001 for STD1, STD2, STF1 and STF2) on activity responses among all network scenarios (*P* < 0.0001 for STD1, STD2, STF1, and STF2) for both mature and immature conditions (*P* < 0.0001 for STD1, STD2, STF1, and STF2). There was an interaction between these three factors (*P* < 0.0001 for STD1, STD2, STF1, and STF2). (2) The 15 Hz IF (beta range) induced a significant effect (*P* < 0.0001 for STD1, STD2, STF1, and STF2) on activity responses among all network scenarios (*P* < 0.0001 for STD1, STD2, STF1, and STF2) for both mature and immature conditions (*P* = 0.0021 for STD1 and *P* < 0.0001 for STD2, STF1, and STF2). There was an interaction between these three factors (*P* < 0.0001 for STD1, STD2, STF1, and STF2). As to the 20 and 30 Hz IF (beta range), they induced a significant effect (*P* < 0.0001 for STD1, STD2, STF1, and STF2) on activity responses among all network scenarios (*P* < 0.0001 for STD1, STD2, STF1 and STF2) for both mature and immature conditions (*P* < 0.0001 for STD1, STD2, STF1, and STF2). There was an interaction between these three factors (*P* < 0.0001 for STD1, STD2, STF1, and STF2). Concerning the 25 Hz IF (beta range), it expressed a significant effect (*P* < 0.0001 for STD1, STD2, STF1, and STF2) on activity responses among all network scenarios (*P* < 0.0001 for STD1, STD2, STF1, and STF2) for both mature and immature conditions (*P* < 0.0001 for STD1, STF1, and STF2 and *P* = 0.0033 for STD2). There was an interaction between these three factors (*P* < 0.0001 for STD1, STD2, STF1, and STF2). (3) As to the 35 and 40 Hz IF (low-gamma range), they induced a significant effect (*P* < 0.0001 for STD1, STD2, STF1, and STF2) on activity responses among all network scenarios (*P* < 0.0001 for STD1, STD2, STF1, and STF2) for both mature and immature conditions (*P* < 0.0001 for STD1, STD2, STF1, and STF2). There was an interaction between these three factors (*P* < 0.0001 for STD1, STD2, STF1, and STF2). As to the 45 Hz IF (low-gamma range), it induced a significant effect (*P* < 0.0001 for STD1, STD2, STF1, and STF2) on activity responses among all network scenarios (*P* < 0.0001 for STD1, STD2, STF1, and STF2) for both mature and immature conditions (*P* < 0.0001 for STD1, STD2, and STF2 while *P* = 0.0148 for STF1). There was an interaction between these three factors (*P* < 0.0001 for STD1, STD2, STF1, and STF2). Regarding the 50 Hz IF (low-gamma range), it induced a significant effect (*P* < 0.0001 for STD1, STD2, STF1, and STF2) on activity responses among all network scenarios (*P* < 0.0001 for STD1, STD2, STF1, and STF2) for both mature and immature conditions (*P* < 0.0001 for STD2 and STF2 while *P* = 0.1635 for STD1 and *P* = 0.0519 for STF1). There was an interaction between these three factors (*P* < 0.0001 for STD1, STD2, STF1, and STF2). (4) For the 55 and 60 Hz IF (high-gamma range), they induced a significant effect (*P* < 0.0001 for STD1, STD2, STF1, and STF2) on activity responses among all network scenarios (*P* < 0.0001 for STD1, STD2, STF1, and STF2) for both mature and immature conditions (*P* < 0.0001 for STD1, STD2, STF1, and STF2). There was an interaction between the three factors (*P* < 0.0001 for STD1, STD2, STF1, and STF2). As to the 65 Hz IF (high-gamma range), it induced a significant effect (*P* < 0.0001 for STD1, STD2, STF1, and STF2) on activity responses among all network scenarios (*P* < 0.0001 for STD1, STD2, STF1, and STF2) for both mature and immature conditions (*P* = 0.0017 for STD2, *P* < 0.0001 for STF1 and *P* = 0.0014 for STF2 while *P* = 0.6643 for STD1). There was an interaction between these three factors (*P* < 0.0001 for STD1, STD2, STF1, and STF2). Concerning the 70 Hz IF (high-gamma range), it induced a significant effect (*P* < 0.0001 for STD1, STD2, STF1, and STF2) on activity responses among all network scenarios (*P* < 0.0001 for STD1, STD2, STF1, and STF2) for both mature and immature conditions (*P* < 0.0001 for STD1, STD2, and STF2 and *P* = 0.0105 for STF1). There was an interaction between these three factors (*P* < 0.0001 for STD1, STD2, STF1, and STF2). As to the 75 Hz IF (high-gamma range), it induced a significant effect (*P* < 0.0001 for STD1, STD2, STF1, and STF2) on activity responses among all network scenarios (*P* < 0.0001 for STD1, STD2, STF1, and STF2) for both mature and immature conditions (*P* < 0.0001 for STD1, STD2, and STF2 while *P* = 0.7598 for STF1). There was an interaction between these three factors (*P* < 0.0001 for STD1, STD2, STF1, and STF2, respectively). Concerning the 80 Hz IF (high-gamma range), it induced a significant effect (*P* < 0.0001 for STD1, STD2, STF1, and STF2) on activity responses among all network scenarios (*P* < 0.0001 for STD1, STD2, STF1, and STF2) for both mature and immature conditions (*P* < 0.0001 for STD1, STF1, and STF2 and *P* = 0.0050 for STD2). There was an interaction between these three factors (*P* < 0.0001 for STD1, STD2, STF1, and STF2). (5) Regarding the 85 and 90 Hz IF (epsilon range), they induced a significant effect (*P* < 0.0001 for STD1, STD2, STF1, and STF2) on activity responses among all network scenarios (*P* < 0.0001 for STD1, STD2, STF1, and STF2) for both mature and immature conditions (*P* < 0.0001 for STD1, STD2, STF1, and STF2). There was an interaction between these three factors (*P* < 0.0001 for STD1, STD2, STF1, and STF2). As to the 95 Hz IF (epsilon range), it induced a significant effect (*P* < 0.0001 for STD1, STD2, STF1, and STF2) on activity responses among all network scenarios (*P* < 0.0001 for STD1, STD2, STF1, and STF2) for both mature and immature conditions (*P* < 0.0001 for STD1 and STF2, *P* < 0.0101 for STF1 while *P* = 0.1470 for STD2). There was an interaction between these three factors (*P* < 0.0001 for STD1, STD2, STF1, and STF2). As to the 100 Hz IF (epsilon range), it induced differential effects (*P* < 0.0001 for STD1, STD2, and STF2 while *P* = 0.0758 for STF1) on activity responses among all network scenarios (*P* < 0.0001 for STD1, STD2, STF1, and STF2) for both mature and immature conditions (*P* < 0.0001 for STD1, STD2, STF1, and STF2). There was an interaction between these three factors (*P* < 0.0001 for STD1, STD2, STF1, and STF2).

**Table 1:** Three-way ANOVA for STD1.

**Ranges**	**IF (Hz)**		**3-Factors**			**Interactions**

			**Control/STD1**	**Networks**	**Maturation**
	Theta	5 Hz	*P* < 0.0001		*P* < 0.0001	*P* < 0.0001
	Alpha	10 Hz			
	Beta	15 Hz			*P* = 0.0021
		20 Hz			*P* < 0.0001
		25 Hz			
		30 Hz			
	Low-Gamma	35 Hz			
		40 Hz			
		45 Hz			
		50 Hz			*P* = 0.1635
	High-Gamma	55 Hz			*P* < 0.0001
		60 Hz			
		65 Hz			*P* = 0.6643
		70 Hz			*P* < 0.0001
		75 Hz			
		80 Hz			
	Epsilon	85 Hz			
		90 Hz			
		95 Hz			
		100 Hz			

**Table 2:** Three-way ANOVA for STD2.

**Ranges**	**IF (Hz)**		**3-Factors**			**Interactions**
			**Control/STD2**	**Networks**	**Maturation**
	Theta	5 Hz	*P* < 0.0001		*P* < 0.0001	*P* < 0.0001
	Alpha	10 Hz			
	Beta	15 Hz			
		20 Hz			
		25 Hz			*P* = 0.0033
		30 Hz			*P* < 0.0001
	Low-Gamma	35 Hz			
		40 Hz			
		45 Hz			
		50 Hz			
	High-Gamma	55 Hz			
		60 Hz			
		65 Hz			*P* = 0.0017
		70 Hz			*P* < 0.0001
		75 Hz			
		80 Hz			*P* = 0.0050
	Epsilon	85 Hz			*P* < 0.0001
		90 Hz			
		95 Hz			*P* = 0.1470
		100 Hz			*P* < 0.0001

**Table 3:** Three-way ANOVA for STF1.

**Ranges**	**IF (Hz)**		**3-Factors**			**Interactions**
			**Control/STF1**	**Networks**	**Maturation**
	Theta	5 Hz	*P* < 0.0001	*P* < 0.0001	*P* < 0.0001	*P* < 0.0001
	Alpha	10 Hz			
	Beta	15 Hz			
		20 Hz			
		25 Hz			
		30 Hz			
	Low-Gamma	35 Hz			
		40 Hz			
		45 Hz			*P* = 0.0148
		50 Hz			*P* = 0.0519
	High-Gamma	55 Hz			*P* < 0.0001
		60 Hz			
		65 Hz			
		70 Hz			*P* = 0.0105
		75 Hz			*P* = 0.7598
		80 Hz			*P* < 0.0001
	Epsilon	85 Hz			
		90 Hz	*P* = 0.0758		
		95 Hz	*P* < 0.0001		*P* < 0.0101
		100 Hz			*P* < 0.0001

**Table 4:** Three-way ANOVA for STF2.

**Ranges**	**IF (Hz)**		**3-Factors**			**Interactions**

			**Control/STF2**	**Networks**	**Maturation**
	Theta	5 Hz	*P* < 0.0001		*P* < 0.0001	*P* < 0.0001
	Alpha	10 Hz		
	Beta	15 Hz			
		20 Hz		
		25 Hz		
		30 Hz		
	Low-Gamma	35 Hz			
		40 Hz			
		45 Hz		
		50 Hz			
	High-Gamma	55 Hz			
		60 Hz			
		65 Hz			*P* = 0.0014
		70 Hz			*P* < 0.0001
		75 Hz		
		80 Hz		
	Epsilon	85 Hz			
		90 Hz			
		95 Hz			
		100 Hz			

In order to monitor the effects of IF on eliciting the response of firing rate activity among all network scenarios of immature and mature network conditions before and after implementing dynamical synapses, we performed a 2-way ANOVA. Then, using the same statistical test (2-way ANOVA), we measured the modulation effects of dynamical synapses (STP) on eliciting the predicted and unpredicted firing rate activity response in each of the network scenarios [(A-1), (A-2), (B-1), (B-2), (B-3), and (B-4)] in immature and mature network conditions. The results of this analysis are shown in Tables 5, 6.

**Table 5:** Two-way ANOVA for immature network scenarios.

**Test**	**Factors**	**Immature network scenarios**					

		**A-1**	**A-2**	**B-1**	**B-2**	**B-3**	**B-4**
Two-way ANOVA	IF (Hz)	*P* = 0.6874	*P* = 0.7235	*P* = 0.0601	*P* = 0.3937	*P* = 0.0420	*P* = 0.1887
	STD1	*P* = 0.0056	*P* = 0.0141	*P* < 0.0001	*P* = 0.4804	*P* = 0.0138	*P* = 0.0010
	Interaction	*P* = 0.2288	*P* = 0.1982	*P* = 0.7052	*P* = 0.3390	*P* = 0.7666	*P* = 0.5381
	IF (Hz)	*P* = 0.3208	*P* = 0.6312	*P* = 0.0048	*P* = 0.0087	*P* = 0.8028	*P* = 0.0593
	E-STD1	*P* = 0.0799	*P* = 0.1182	*P* < 0.0001	*P* = 0.0003	*P* = 0.2771	*P* < 0.0001
	Interaction	*P* = 0.4340	*P* = 0.2621	*P* = 0.9724	*P* = 0.7322	*P* = 0.1295	*P* = 0.6967
	IF (Hz)	*P* = 0.1178	*P* = 0.1755	NA	*P* = 0.7046	*P* = 0.0001	*P* = 0.3960
	U-STD1	*P* = 0.0914	*P* = 0.0361		*P* = 0.0084	*P* = 0.0027	*P* = 0.1381
	Interaction	*P* = 0.7134	*P* = 0.6348		*P* = 0.2789	*P* = 0.9991	*P* = 0.3676
	IF (Hz)	*P* = 0.6494	*P* = 0.4746	*P* = 0.8887	*P* = 0.1572	*P* = 0.0419	*P* = 0.1704
	STD2	*P* = 0.0360	*P* = 0.0143	*P* = 0.0143	*P* = 0.4192	*P* = 0.0328	*P* = 0.0008
	Interaction	*P* = 0.2314	*P* = 0.3445	*P* = 0.1275	*P* = 0.5573	*P* = 0.7679	*P* = 0.5055
	IF (Hz)	*P* = 0.1542	*P* = 0.1500	*P* = 0.0743	*P* = 0.0011	*P* = 0.3464	*P* = 0.1387
	E-STD2	*P* = 0.0034	*P* = 0.0001	*P* < 0.0001	*P* = 0.0002	*P* = 0.1183	*P* < 0.0001
	Interaction	*P* = 0.6503	*P* = 0.5659	*P* = 0.8572	*P* = 0.9767	*P* = 0.3590	*P* = 0.4930
	IF (Hz)	*P* = 0.1698	*P* = 0.0609	*P* = 0.6451	*P* = 0.5167	*P* = 0.0581	*P* = 0.0707
	U-STD2	*P* = 0.1183	*P* = 0.0379	*P* = 0.0185	*P* = 0.0254	*P* = 0.0216	*P* = 0.0342
	Interaction	*P* = 0.6369	*P* = 0.8210	*P* = 0.2755	*P* = 0.3415	*P* = 0.8273	*P* = 0.8146
	IF (Hz)	*P* = 0.3753	*P* = 0.2654	*P* = 0.0268	*P* < 0.0001	*P* = 0.1606	*P* = 0.0930
	STF1	*P* = 0.5383	*P* = 0.2200	*P* = 0.0047	*P* = 0.0573	*P* = 0.4650	*P* = 0.0127
	Interaction	*P* = 0.3955	*P* = 0.4702	*P* = 0.7639	*P* = 0.9946	*P* = 0.5654	*P* = 0.6354
	IF (Hz)	*P* = 0.2267	*P* = 0.0179	*P* = 0.0525	*P* = 0.1880	*P* = 0.0743	*P* = 0.2811
	E-STF1	*P* = 0.0584	*P* = 0.0179	*P* = 0.1024	*P* = 0.0927	*P* = 0.0055	*P* = 0.1006
	Interaction	*P* = 0.5569	*P* = 0.9220	*P* = 0.8578	*P* = 0.6056	*P* = 0.7904	*P* = 0.4978
	IF (Hz)	*P* = 0.0174	*P* = 0.2269	*P* = 0.0135	*P* = 0.0211	*P* = 0.0023	*P* = 0.6738
	U-STF1	*P* = 0.0726	*P* = 0.0238	*P* = 0.0008	*P* = 0.0156	*P* = 0.0006	*P* = 0.0649
	Interaction	*P* = 0.9495	*P* = 0.5593	*P* = 0.8580	*P* = 0.9234	*P* = 0.9431	*P* = 0.9474
	IF (Hz)	*P* = 0.3149	*P* = 0.1633	*P* = 0.0273	*P* = 0.0040	*P* = 0.0031	*P* = 0.0515
	STF2	*P* = 0.1536	*P* = 0.0464	*P* = 0.0057	*P* = 0.7882	*P* = 0.2416	*P* = 0.0031
	Interaction	*P* = 0.4339	*P* = 0.5158	*P* = 0.7963	*P* = 0.9285	*P* = 0.9442	*P* = 0.7100
	IF (Hz)	*P* = 0.0234	*P* = 0.0296	*P* = 0.0076	*P* = 0.2728	*P* = 0.0027	*P* = 0.0036
	E-STF2	*P* = 0.0233	*P* = 0.1868	*P* = 0.0165	*P* = 0.0024	*P* = 0.0212	*P* = 0.0203
	Interaction	*P* = 0.9078	*P* = 0.8899	*P* = 0.9730	*P* = 0.5459	*P* = 0.9814	*P* = 0.9844
	IF (Hz)	*P* = 0.2578	*P* = 0.1575	*P* = 0.0036	*P* = 0.0086	*P* = 0.0036	*P* = 0.5326
	U-STF2	*P* = 0.3356	*P* = 0.0048	*P* < 0.0001	*P* = 0.0139	*P* = 0.0030	*P* = 0.2930
	Interaction	*P* = 0.4749	*P* = 0.6330	*P* = 0.9581	*P* = 0.9650	*P* = 0.9632	*P* = 0.3404

**Table 6:** Two-way ANOVA for mature network scenarios.

**Test**	**Factors**	**Mature network scenarios**					

		**A-1**	**A-2**	**B-1**	**B-2**	**B-3**	**B-4**
Two-way ANOVA	IF (Hz)	*P* = 0.0286	*P* = 0.4593	*P* = 0.0126	*P* = 0.4481	*P* = 0.0744	*P* = 0.2225
	STD1	*P* = 0.3141	*P* = 0.0438	*P* = 0.0001	*P* = 0.0025	*P* = 0.0144	*P* = 0.0050
	Interaction	*P* = 0.9024	*P* = 0.6237	*P* = 0.9485	*P* = 0.5810	*P* = 0.8062	*P* = 0.6382
	IF (Hz)	*P* < 0.0001	*P* = 0.0083	*P* = 0.0043	*P* = 0.1155	*P* = 0.0014	*P* = 0.0301
	E-STD1	*P* = 0.0004	*P* < 0.0001	*P* < 0.0001	*P* < 0.0001	*P* < 0.0001	*P* < 0.0001
	Interaction	*P* = 0.9963	*P* = 0.9058	*P* = 0.9066	*P* = 0.6133	*P* = 0.9655	*P* = 0.8062
	IF (Hz)	*P* = 0.3463	*P* = 0.9960	*P* = 0.0139	*P* = 0.1960	*P* = 0.4269	*P* = 0.0415
	U-STD1	*P* = 0.1075	*P* = 0.0694	*P* = 0.1440	*P* = 0.1510	*P* = 0.1435	*P* = 0.0286
	Interaction	*P* = 0.1376	*P* = 0.2410	*P* = 0.8160	*P* = 0.1510	*P* = 0.1871	*P* = 0.7094
	IF (Hz)	*P* < 0.0001	*P* = 0.0723	*P* = 0.0237	*P* = 0.0439	*P* = 0.0490	*P* = 0.0064
	STD2	*P* = 0.0002	*P* = 0.2081	*P* = 0.0004	*P* < 0.0001	*P* = 0.0101	*P* = 0.0027
	Interaction	*P* = 0.9990	*P* = 0.9015	*P* = 0.9244	*P* = 0.9418	*P* = 0.8665	*P* = 0.9583
	IF (Hz)	*P* < 0.0001	*P* = 0.0008	*P* = 0.0109	*P* = 0.0439	*P* = 0.1250	*P* = 0.0011
	E-STD2	*P* < 0.0001	*P* = 0.0004	*P* < 0.0001	*P* < 0.0001	*P* < 0.0001	*P* < 0.0001
	Interaction	*P* = 0.9993	*P* = 0.9803	*P* = 0.8694	*P* = 0.8076	*P* = 0.5476	*P* = 0.9756
	IF (Hz)	*P* = 0.0035	*P* = 0.3276	*P* = 0.0022	NA	*P* = 0.0070	*P* = 0.2389
	U-STD2	*P* = 0.0330	*P* = 0.1453	*P* = 0.0323		*P* = 0.1185	*P* = 0.1024
	Interaction	*P* = 0.9973	*P* = 0.3188	*P* = 0.9896		*P* = 0.9244	*P* = 0.3120
	IF (Hz)	*P* = 0.0710	*P* = 0.0084	*P* = 0.0002	*P* = 0.0337	*P* = 0.0035	*P* = 0.0049
	STF1	*P* = 0.9783	*P* = 0.4688	*P* = 0.0042	*P* = 0.0275	*P* = 0.0081	*P* = 0.0009
	Interaction	*P* = 0.8885	*P* = 0.9781	*P* = 0.9951	*P* = 0.9072	*P* = 0.9738	*P* = 0.9408
	IF (Hz)	*P* = 0.0205	*P* = 0.0507	*P* = 0.0364	*P* = 0.0383	*P* = 0.4342	*P* = 0.1879
	E-STF1	*P* = 0.0170	*P* = 0.0990	*P* = 0.0275	*P* = 0.1354	*P* = 0.0806	*P* = 0.0835
	Interaction	*P* = 0.7981	*P* = 0.6659	*P* = 0.6396	*P* = 0.7059	*P* = 0.1271	*P* = 0.7722
	IF (Hz)	*P* = 0.0371	*P* = 0.0043	*P* < 0.0001	*P* = 0.0196	*P* = 0.0005	*P* = 0.0008
	U-STF1	*P* = 0.0048	*P* = 0.0018	*P* < 0.0001	*P* = 0.0023	*P* < 0.0001	*P* < 0.0001
	Interaction	*P* = 0.8118	*P* = 0.9637	*P* = 0.9972	*P* = 0.8810	*P* = 0.9867	*P* = 0.9919
	IF (Hz)	*P* = 0.5415	*P* = 0.0551	*P* = 0.0002	*P* = 0.6318	*P* = 0.0015	*P* = 0.0402
	STF2	*P* = 0.6990	*P* = 0.6555	*P* = 0.0011	*P* = 0.4395	*P* = 0.0059	*P* = 0.2029
	Interaction	*P* = 0.4893	*P* = 0.9237	*P* = 0.9976	*P* = 0.4845	*P* = 0.9864	*P* = 0.8438
	IF (Hz)	*P* = 0.1957	*P* = 0.0949	*P* = 0.0028	*P* = 0.0513	*P* = 0.0003	*P* = 0.1066
	E-STF2	*P* = 0.0285	*P* = 0.0255	*P* = 0.0550	*P* = 0.0069	*P* = 0.0572	*P* = 0.0156
	Interaction	*P* = 0.4608	*P* = 0.5346	*P* = 0.9397	*P* = 0.6990	*P* = 0.9772	*P* = 0.5089
	IF (Hz)	*P* = 0.3056	*P* = 0.0111	*P* < 0.0001	*P* = 0.2767	*P* = 0.0031	*P* = 0.0110
	U-STF2	*P* = 0.0008	*P* < 0.0001	*P* < 0.0001	*P* = 0.0030	*P* = 0.0002	*P* = 0.0006
	Interaction	*P* = 0.4353	*P* = 0.8867	*P* = 0.9961	*P* = 0.4563	*P* = 0.9586	*P* = 0.9414

#### Measures of MSF and Predicting a Developmental Shift

We eliminated all unfitted responses that revealed significant variations in the scale of probability density function (PDF) of the Gaussian distribution curve of MSF (see Figures 3–17 of Khalil et al., 2017b). Therefore, we interpreted only data that did not show statistical fluctuations in PDF, before and after implementing STP.

## Results

The current study is an extension of our prior plasticity modeling study (Khalil et al., 2017a,b). Here, we provide a quantitative analysis of the relationship between the modulator roles of a particular external signaling element and other crucial intrinsic signaling factors, and their influence on the maintenance of neural activity [which might be experimentally observed in **M**ulti**-E**lectrode **A**rray**s** (**MEAs**)]. The external signal element is represented in STP (triggered by different ranges of IF). On the other hand, the depolarizing and hyperpolarizing state of GABA_A_ signaling, a local density of dendritic arborization [i.e., lateral connectivity (distance between adjacent neurons)] and a lateral spread length between neighbor neurons [i.e., the nearby local connectivity (complex connections involving those between neuronal groups)] refer to other crucial intrinsic signaling factors.

### Dynamical Synapses and Modulation of Neural Network Activity Through Synaptic Fine-Tuning in Two Physiological States of GABA_A_

This study shows our simulation results before and after dynamically varying the synaptic status (i.e., after implementing STP). We monitored the firing rate activity (Hz) during 2,000 s in all neural networks in both conditions, immature and mature network. Our 2-way ANOVA, conducted as a follow up test after 3-way ANOVA, observed the effects of IF on eliciting firing rate activity response among all network scenarios [(A-1), (A-2), (B-1), (B-2), (B-3), and (B-4)] of immature and mature network conditions before and after implementing dynamical synapses (STD1, STD2, STF1, and STF2). It revealed the following results: (1) for the immature condition, there was no significant effect for IF (*P* = *0.0566*) on eliciting the response of firing rate activity among all network scenarios (*P* = *0.3586*), there was no interaction between IF and network scenarios as well (*P* = *0.4928*). However, implementing short-term depressing synapses revealed only a significant effect of IF (*P* = *0.0089* and *P* = *0.0113* for STD1 and STD2, respectively) on the activity responses among all network scenarios (*P* = *0.6512* and *P* = *0.5675* for STD1 and STD2, respectively) and there was no interaction between IF and network scenarios (*P* = *0.5025* and *P* = *0.4903* for STD1 and STD2, respectively). In contrast, implementing STF did not show any significant effect of IF (*P* = *0.0508* and *P* = *0.2868* for STF1 and STF2, respectively) on the activity responses among all network scenarios (*P* = *0.4164* and *P* = *0.1524* for STF1 and STF2, respectively) and there was no interaction between IF and network scenarios (*P* = *0.6431* and *P* = *0.2064* for STF1 and STF2, respectively). (2) The mature condition revealed a different result in comparison to the immature condition. For instance, before implementing STP, there was a significant effect only for IF (*P* = *0.0389*) on eliciting firing rate activity response among all network scenarios (*P* = *0.7118*), there was no interaction as well between IF and network scenarios (*P* = *0.9963*). In contrast with immature conditions, STD in mature conditions did not show any significant effect of IF (*P* = *0.1037* and *P* = *0.1148* for STD1 and STD2, respectively), on activity responses among all network scenarios (*P* = *0.1640* and *P* = *0.7020* for STD1 and STD2, respectively). There was no interaction between IF and network scenarios (*P* = *0.7911* and *P* = *0.5521* for STD1 and STD2, respectively). In contrast with depressing synapses, facilitating synapses in mature conditions revealed significant effects of IF (*P* = *0.0094* and *P* = *0.0086* for STF1 and STF2, respectively) on activity responses among all network scenarios (*P* = *0.4270* and *P* = *0.6037* for STF1 and STF2, respectively). There was no interaction between IF and network scenarios (*P* = *0.9464* and *P* = *0.9965* for STF1 and STF2, respectively). Overall, this observation indicates that there are differential effects of IF and dynamical synapses- triggered by- IF on modulating firing rate responses, which varies in both conditions (immature and mature ones). Concerning the IF, each class of STP (i.e., STD and STF) revealed significant reversal effect in eliciting modulating responses of the firing rate activity based on the condition of maturation reflecting the physiological state of GABA_A_ (i.e., immature and mature conditions). In addition, there was a preference toward selecting particular ranges of IF to be triggered by dynamical synapses in each physiological state of GABA_A_ (Figure 1). Subsequently, we segregated the results into two sections according to modulating effects induced by STP.

### Modulated Responses Through Two Physiological States of GABA_A_

#### Predicted and Unpredicted Modulated Responses

**Figure 2:**
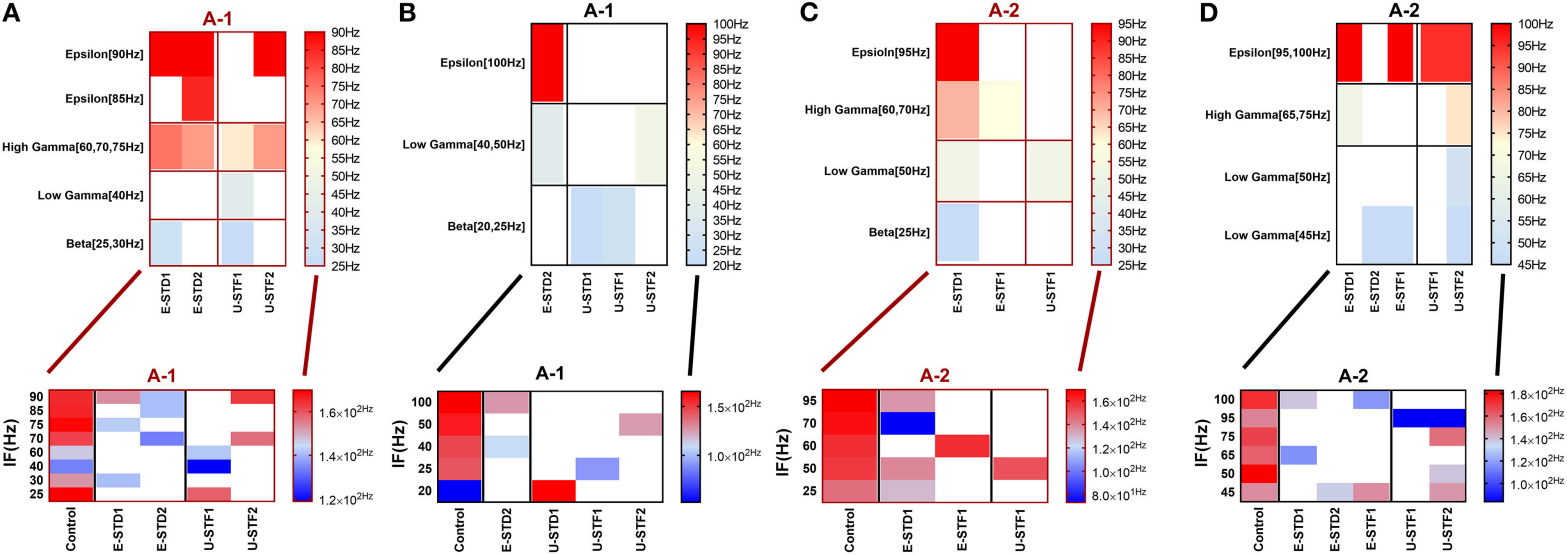
Heat maps of selected elicited responses in each network scenario (A-1) and (A-2), in immature and mature conditions, respectively, before and after implementing dynamical synapses. **(A,C)** refer to immature conditions of network scenarios (A-1 and A-2) while **(B,D)** relate to mature conditions of network scenarios (A-1 and A-2). The upper graphs refer to particular Poisson input frequencies [IF (Hz)] that triggered a substantial modulation effect, measured in Hz before and after STP in these networks (Lower graphs). White blocks refer to excluded values due to statistical variations in probability density function (PDF). Control refers to the condition without STP implementation (dynamical synapses); STD1 and STD2 refer to two types of depressing synapses while STF1 and STF2 refer to facilitating synapse. E-STD1, E-STD1, E-STF1, and E-STF2 relate to the expected modulation effect induced by dynamical synapses i.e., reduction of firing rate activities in response to depressing synapses (STD1 and STD2) while increasing the firing rate activity in response to facilitating synapses (STF1 and STF2). On the other hand, U-STD1, U-STD1, U-STF1, and U-STF2 relate to the expected modulation effect induced by dynamical synapses, i.e., an increase of firing rate activity in response to depressing synapses (STD1 and STD2), while reduction in the firing rate activity in response to facilitating synapses (STF1 and STF2), see also Tables 5, 6.

**Figure 3:**
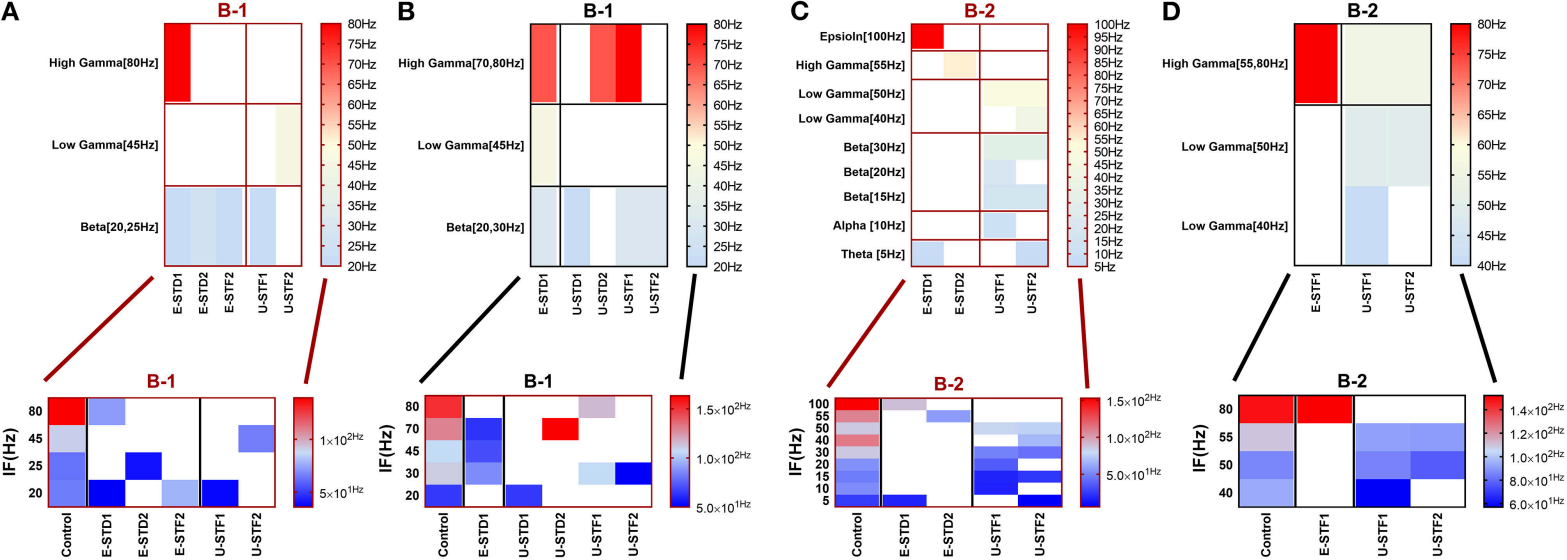
Heat maps of selected elicited responses in each network scenario (B-1) and (B-2), in both immature and mature conditions, respectively, before and after implementing dynamical synapses. **(A,C)** refer to immature conditions of network scenarios (B-1 and B-2) while **(B,D)** relate to mature conditions of network scenarios (B-1 and B-2). The upper graphs refer to particular Poisson input frequencies [IF (Hz)] that triggered a substantial modulation effect, measured in Hz before and after STP in these networks (Lower graphs). White blocks refer to excluded values due to statistical variations in probability density function (PDF). Control refers to the condition without STP implementation (dynamical synapses); STD1 and STD2 refer to two types of depressing synapses while STF1 and STF2 refer to facilitating synapses. E-STD1, E-STD1, E-STF1, and E-STF2 relate to the expected modulation effect induced by dynamical synapses, i.e., reduction of firing rate activities in response to depressing synapses (STD1 and STD2) while increasing the firing rate activity in response to facilitating synapses (STF1 and STF2). On the other hand, U-STD1, U-STD1, U-STF1, and U-STF2 relate to the expected modulation effect induced by dynamical synapses, i.e., an increase of firing rate activities in response to depressing synapses (STD1 and STD2), while reduction in the firing rate activity in response to facilitating synapses (STF1 and STF2), see also Tables 5, 6.

The influence of dynamical synapses on eliciting a significant predicted and unpredicted modulated firing activity, is expressed uniquely among network scenarios (A-1), (A-2), (B-1), (B-2), (B-3), and (B-4), see Tables 5, 6. Predicted (i.e., expected) modulation effect implies a reduction in the firing rate activity in case of STD1 and STD2, and a reverse effect in case of STF1 and STF2. Unpredicted (i.e., unexpected) modulation effect suggests an increase in the firing rate activity in case of STD1 and STD2 and an opposing effect in case of STF1 and STF2. Additionally, these variations were not only observed in network scenarios but also in each condition (immature and mature). We presented the modulated responses in Figures 2, 3, 12.

#### Predicted Modulated Responses

In response to depressing synapses (STD1 and STD2), the significant level of the predicted (expected) elicited modulated effect induced by STD1 was observed both in immature and mature conditions, within their respective network scenarios. For instance, the significance of the expected reduction provoked by STD1 (i.e., E-STD1), in immature conditions, was revealed by a two-way ANOVA as follows (see also Tables 5, 6): the IF (Hz) induced significant effects (*P* = 0.0048 for B-1 and *P* = 0.0087 for B-2), as well as E-STD1 that expressed significant effects (*P* < 0.0001 for B-1, *P* = 0.0003 for B-2 and *P* < 0.0001 for B-4). Variously, for mature network condition, IF (Hz) induced significant effects [*P* < 0.0001(A-1), *P* = 0.0083 (A-2), *P* = 0.0043 (B-1), *P* = 0.0014 (B-3) and *P* = 0.0301(B-4)] while E-STD1 expressed significant effects [*P* = 0.0004(A-1), *P* < 0.0001(A-2), *P* < 0.0001(B-1), *P* < 0.0001(B-2), *P* < 0.0001(B-3), and *P* < 0.0001(B-4)]. In response to STD2, the significant level of the predicted elicited modulated effect induced by STD2 (i.e., E-STD2) was observed in both immature and mature conditions, within their network scenarios. Through the immature network condition, IF (Hz) induced a significant effect [*P* = 0.0011 (B-2)], while E-STD2 expressed significant effects [*P* = 0.0034 (A-1), *P* = 0.0001(A-2), *P* < 0.0001(B-1), *P* = 0.0002 (B-2), and *P* < 0.0001(B-4)]. In contrast, for the mature condition, IF (Hz) induced significant effects [*P* < 0.0001(A-1), *P* = 0.0008 (A-2), *P* = 0.0109 (B-1), *P* = 0.0439 (B-2), and *P* = 0.0011(B-4)], while E-STD2 expressed significant effects [*P* < 0.0001 (A-1), *P* = 0.0004 (A-2), *P* < 0.0001 for (B-1), (B-2), (B-3), and (B-4)]. In case of facilitating synapses (STF1 and STF2), the significance of predicted modulated effects induced by STF1 and STF2, in both immature and mature conditions, within their respective network scenarios is illustrated as follows. In immature conditions, IF (Hz) induced a significant effect [*P* = 0.0179 (A-2)], as well as E-STF1 that expressed significant effects [*P* = 0.0179 (A-2) and *P* = 0.0055 (B-3)]. However, E-STF2 expressed significant effects [*P* = 0.0233 (A-1), *P* = 0.0165 (B-1), *P* = 0.0024 (B-2), *P* = 0.0212 (B-3), and *P* = 0.0203 (B-4)], while IF (Hz) induced significant effects [*P* = 0.0234 (A-1), *P* = 0.0296 (A-2), *P* = 0.0076 (B-1), *P* = 0.0027(B-3), and *P* = 0.0036 (B-4)]. Regarding mature conditions, E-STF1 expressed significant effects [*P* = 0.0170 (A-1) and *P* = 0.0275 (B-1)] and IF (Hz) induced significant effects [*P* = 0.0205 (A-1), *P* = 0.0364 (B-1) and *P* = 0.0383 (B-2)]. Nevertheless, E-STF2 expressed significant effects [*P* = 0.0285 (A-1), *P* = 0.0255 (A-2), *P* = 0.0069 (B-2), and *P* = 0.0156 (B-4)], while IF (Hz) induced significant effects [*P* = 0.0028 (B-1) and *P* = 0.0003 (B-3)].

#### Unpredicted Modulated Responses

In response to depressing synapses (STD1 and STD2), the significant level of the unpredicted (unexpected) elicited modulated effect induced by STD1 (i.e., U-STD1) was observed in the immature condition as follows; U-STD1 expressed significant effects [*P* = 0.0361 (A-2), *P* = 0.0084 (B-2) and *P* = 0.0027 (B-3)], while IF (Hz) induced a significant effect [*P* = 0.0001 (B-3)]. In the case of a mature condition, IF (Hz) induced significant effects [*P* = 0.0139 (B-1) and *P* = 0.0415 (B-4)], while U-STD1 expressed a significant effect [*P* = 0.0286 (B-4)]. As for the significant level of the unpredicted elicited modulated effect induced by STD2 (i.e., U-STD2), it was observed in both immature and mature conditions, within their particular network scenarios. For example, in immature condition, IF (Hz) did not induce any significant effect at any network scenario, however, U-STD2 expressed significant effects [*P* = 0.0379 (A-2), *P* = 0.0185 (B-1), *P* = 0.0254 (B-2), *P* = 0.0216(B-3), and *P* = 0.0342(B-4)]. In comparison, the mature condition revealed the following: IF (Hz) induced significant effects on (A-1), (B-1), and (B-3) (*P* = 0.0035, *P* = 0.0022, and *P* = 0.0070, respectively), but U-STD2 expressed significant effects [*P* = 0.0330 (A-1) and *P* = 0.0323 (B1)]. Concerning facilitating synapses (STF1 and STF2), the significant level of the unpredicted elicited modulated effect induced by STF1 was observed in both immature and mature conditions, within their particular network scenarios. In case of the immature condition, IF (Hz) induced significant effects [*P* = 0.0174 (A-1), *P* = 0.0135(B-1), *P* = 0.0211(B-2), and *P* = 0.0023 (B-3)], while U-STF1 expressed significant effects [*P* = 0.0238 (A-2), *P* = 0.0008 (B-1), *P* = 0.0156 (B-2), and *P* = 0.0006 (B-3)]. For the mature condition, IF (Hz) induced significant effects [*P* = 0.0371(A-1), *P* = 0.0043(A-2), *P* < 0.0001(B-1), *P* = 0.0196(B-2), *P* = 0.0005(B-3), and *P* = 0.0008(B-4)], while U-STF1 expressed significant effects [*P* = 0.0048(A-1), *P* = 0.0018(A-2), *P* < 0.0001(B-1), *P* = 0.0023(B-2), *P* < 0.0001(B-3), and *P* < 0.0001(B-4)].

Regarding the unpredicted modulation effects induced by STF2 in the immature condition, IF (Hz) induced significant effects [*P* = 0.0036(B-1), *P* = 0.0086 (B-2), and *P* = 0.0036 (B-3)], while U-STF2 expressed significant effects [*P* = 0.0048 (A-2), *P* < 0.0001(B-1), *P* = 0.0139 (B-2), and *P* = 0.0030 (B-3)]. As for the mature condition, IF (Hz) induced significant effects [*P* = 0.0111(A-2), *P* < 0.0001 (B-1) *P* = 0.0031 (B-3), and *P* = 0.0110 (B-4)], while U-STF2 expressed significant effects [*P* = 0.0008 (A-1), *P* < 0.0001 (A-2), *P* < 0.0001 (B-1), *P* = 0.0030 (B2), *P* = 0.0002 (B-3), and *P* = 0.0006 (B-4)].

### Prediction of a Developmental Shift

We observed a trend toward modulating the neural firing activity of immature network scenarios through expressing significant changes in the amount of mean spike frequency (MSF) in response to STP (Figures 2A,C, 3A,C, 12A,C). This trend might resemble what could be experimentally recorded in MEAs during early developmental stages *in vitro* [before (5–10 DIV) and during (11–17 DIV) of the GABA_A_ shift, i.e., the change in GABAergic synaptic transmission]. Such modulation might predict a developmental shift to an earlier time window that could be within or before the same stage (i.e., for the mature condition, see Khalil et al., 2017b, P12–P16).

### A Developmental Shift to an Earlier Time Window, Before the First Week *in vitro* (5–10 DIV)

**Figure 4:**
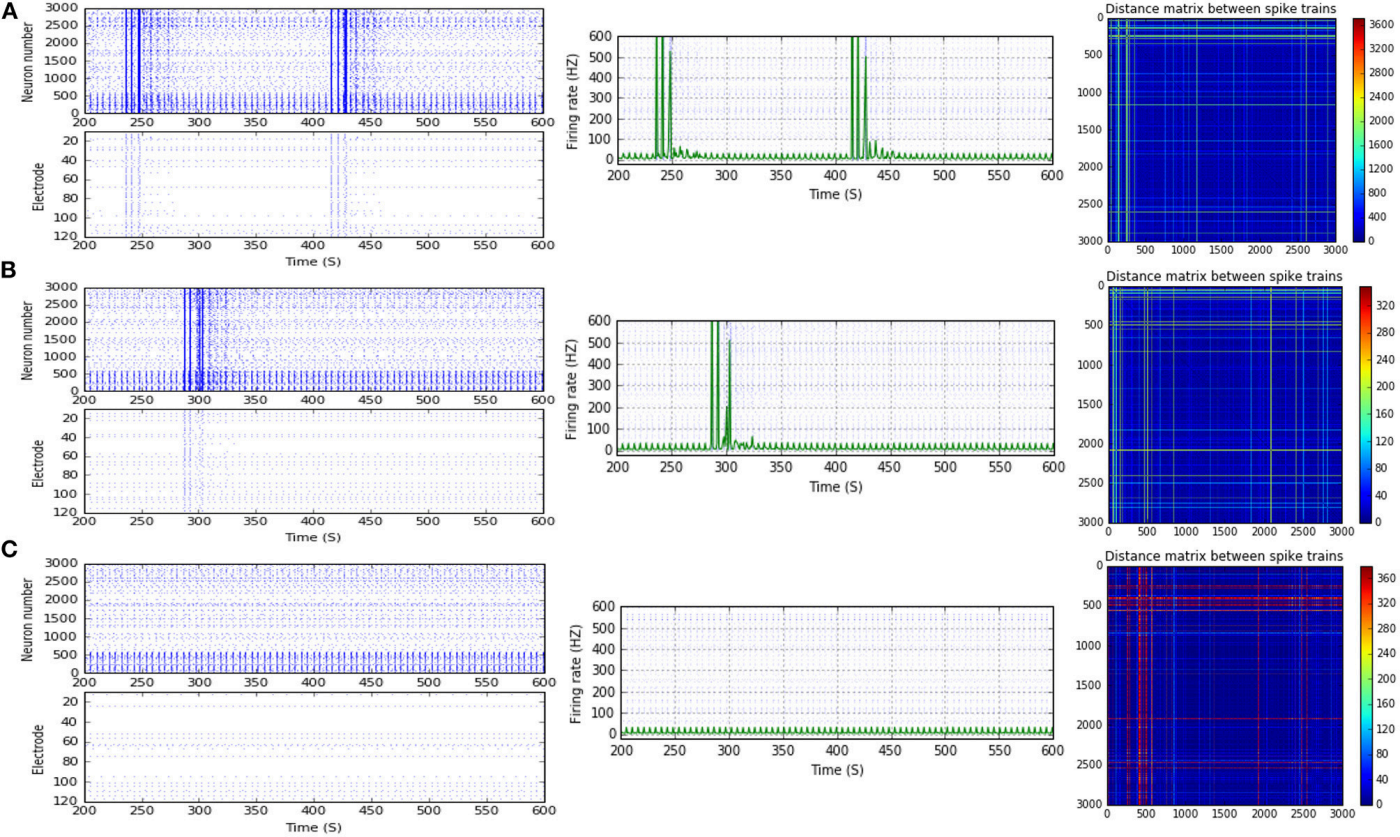
Selected elicited firing response activity in the immature network scenario (B-2) before and after implementing STD1 and STF2 (when IF = 5 Hz: in the range of theta). **(A)** refers to the condition without implementing dynamical synapses (control) while **(B,C)** refer to that after implementing depressing synapses (STD1) and facilitating synapses (STF2), respectively. The first two figures of each panel refer to monitoring the pattern of firing activity in time windows from 200 to 600 seconds (s). Heat maps of each panel represent modulation changes in spike distance matrix of the network scenario (B-2) before and after implementing STD1 and STF2. The simulation time for heat maps of the spike distance is equal to 2,000 s.

In response to STF2 (when IF = 5 Hz: in the range of theta), (B-2) expresses a remarkable reduction in MSF amount (about 76.7%, from 25.8 to 6 Hz; Figures 3C, 4A,C). This might suggest a dynamical developmental shift in the firing activity, which might be observed experimentally *in vitro* (in MEAs) in early stages (before (5–10 DIV). This amount of MSF was experimentally reported to be in the range of 7.86 ± 1.30 Hz at this stage (Table 1; Baltz et al., 2010).

### A Developmental Shift to the Transitional Period Between Week 1 and 2 *in vitro* [Before (5–10 DIV) and During (11–17 DIV) of the GABA_A_ Shift]

**Figure 5:**
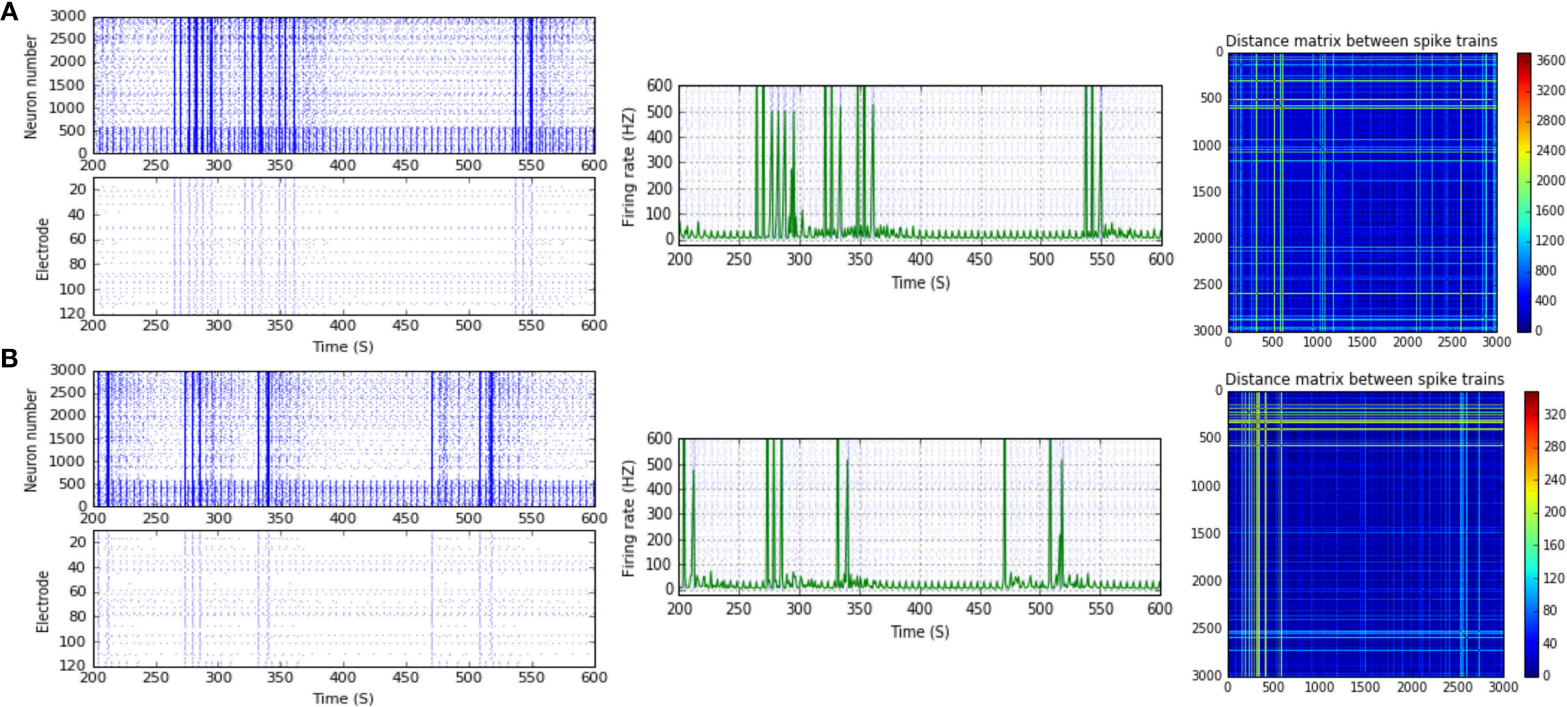
Selected elicited firing response activity in the immature network scenario (B-2) before and after implementing STF1 (when IF = 10 Hz: in the range of alpha). **(A)** refers to the condition without implementing dynamical synapses (control) while **(B)** refers to that after implementing facilitating synapses (STF1). The first two figures of each panel refer to monitoring the pattern of firing activity in time windows from 200 to 600 seconds (s). Heat maps of each panel represent modulation changes in spike distance matrix of the network scenario (B-2) before and after implementing STF1. The simulation time for heat maps of the spike distance is equal to 2,000 s.

**Figure 6:**
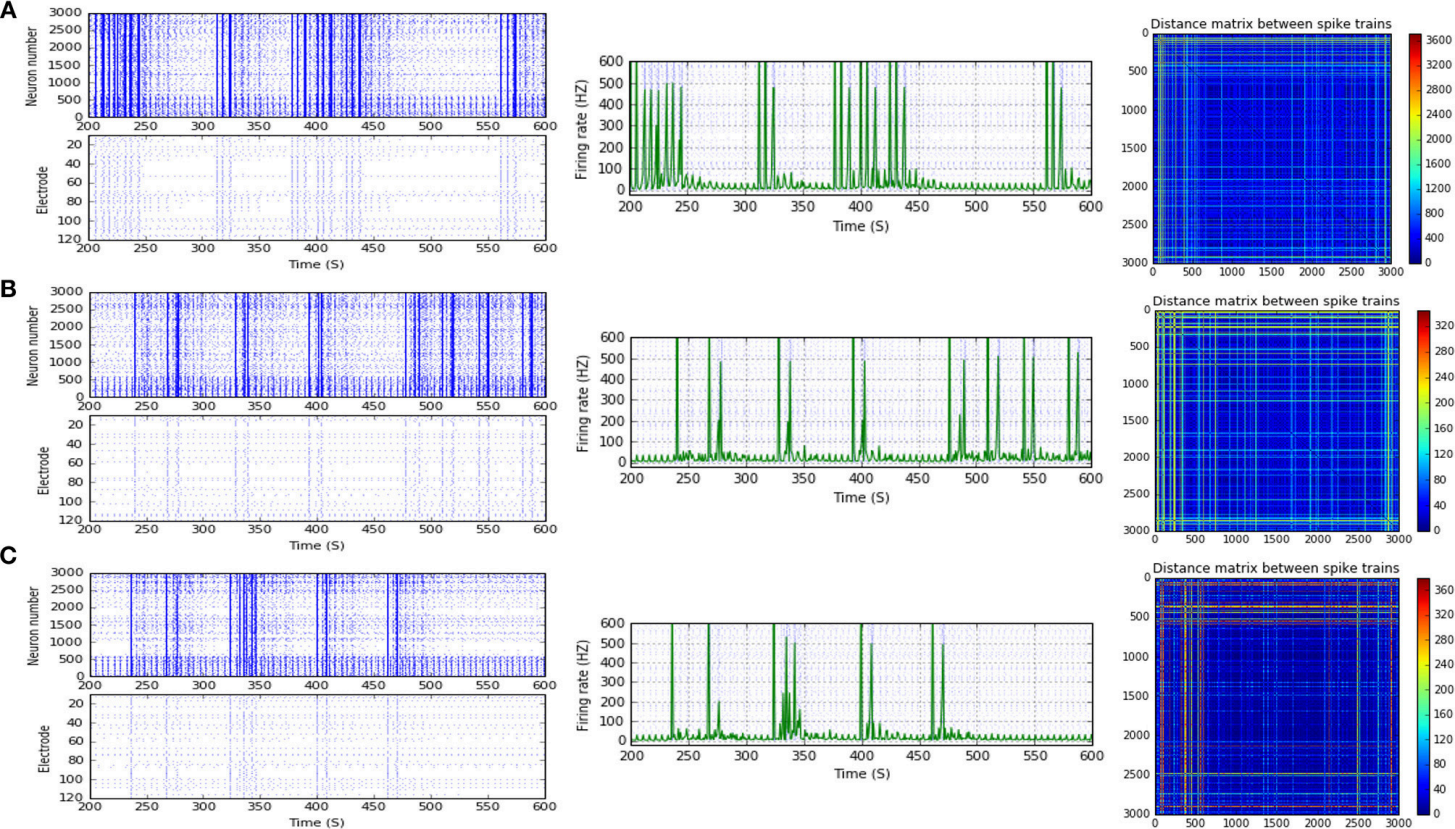
Selected elicited firing response activity in the immature network scenario (B-2) before and after implementing STF1 and STF2 (when IF = 15 Hz: in the range of beta). **(A)** refers to the condition without implementing dynamical synapses (control) while **(B,C)** refer to that after implementing facilitating synapses (STF1 and STF2, respectively). The first two figures of each panel refer to monitoring the pattern of firing activity in time windows from 200 to 600 seconds(s). Heat maps of each panel represent modulation changes in spike distance matrix of the network scenario (B-2) before and after implementing STD1 and STF2. The simulation time for heat maps of the spike distance is equal to 2,000 s.

**Figure 7:**
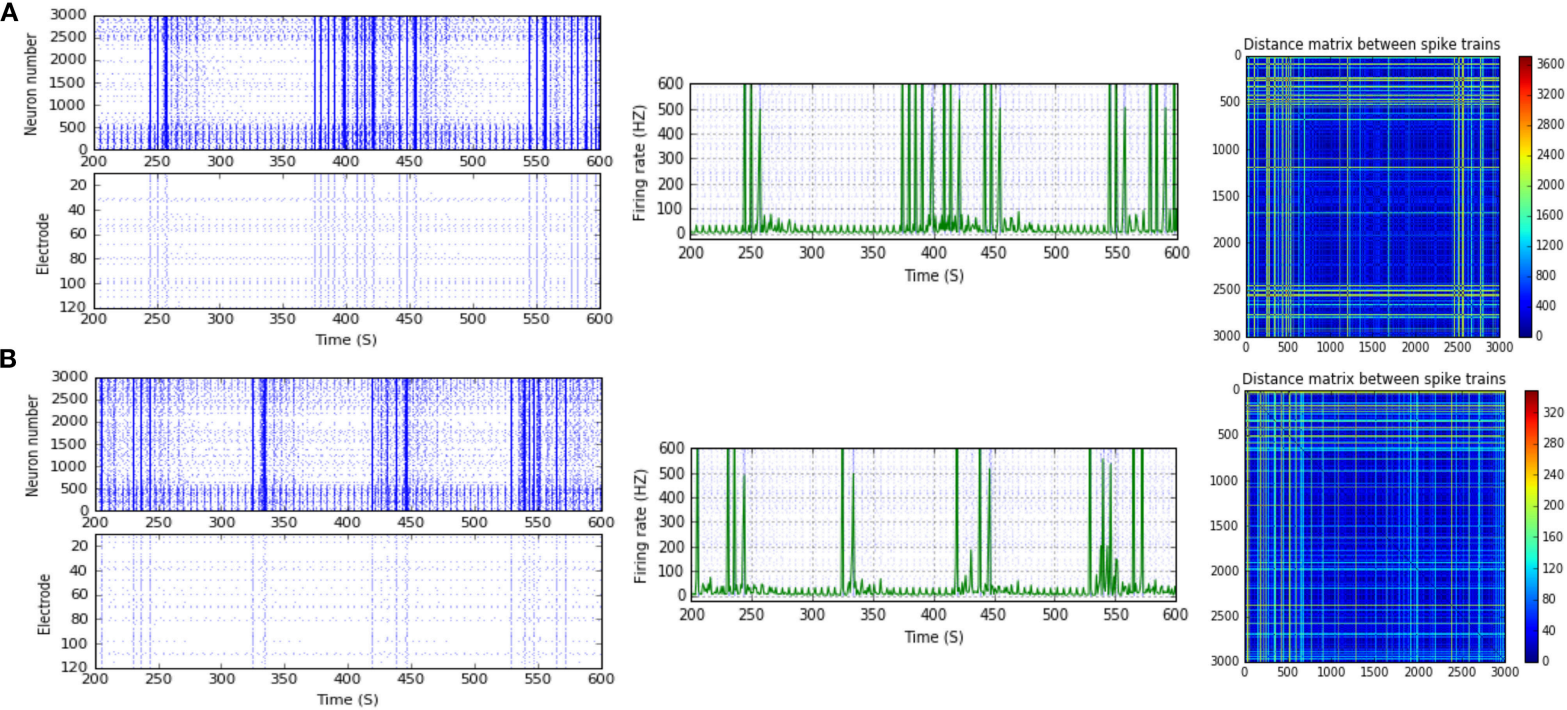
Selected elicited firing response activity in the immature network scenario (B-2) before and after implementing STF1 (when IF = 20 Hz: in the range of beta). **(A)** refers to the condition without implementing dynamical synapses (control) while **(B)** refers to that after implementing facilitating synapses (STF1). The first two figures of each panel refer to monitoring the pattern of firing activity in time windows from 200 to 600 seconds(s). Heat maps of each panel represent the modulation changes in spike distance matrix of the network scenario (B-2) before and after implementing STF1. The simulation time for heat maps of the spike distance is equal to 2,000 s.

**Figure 8:**
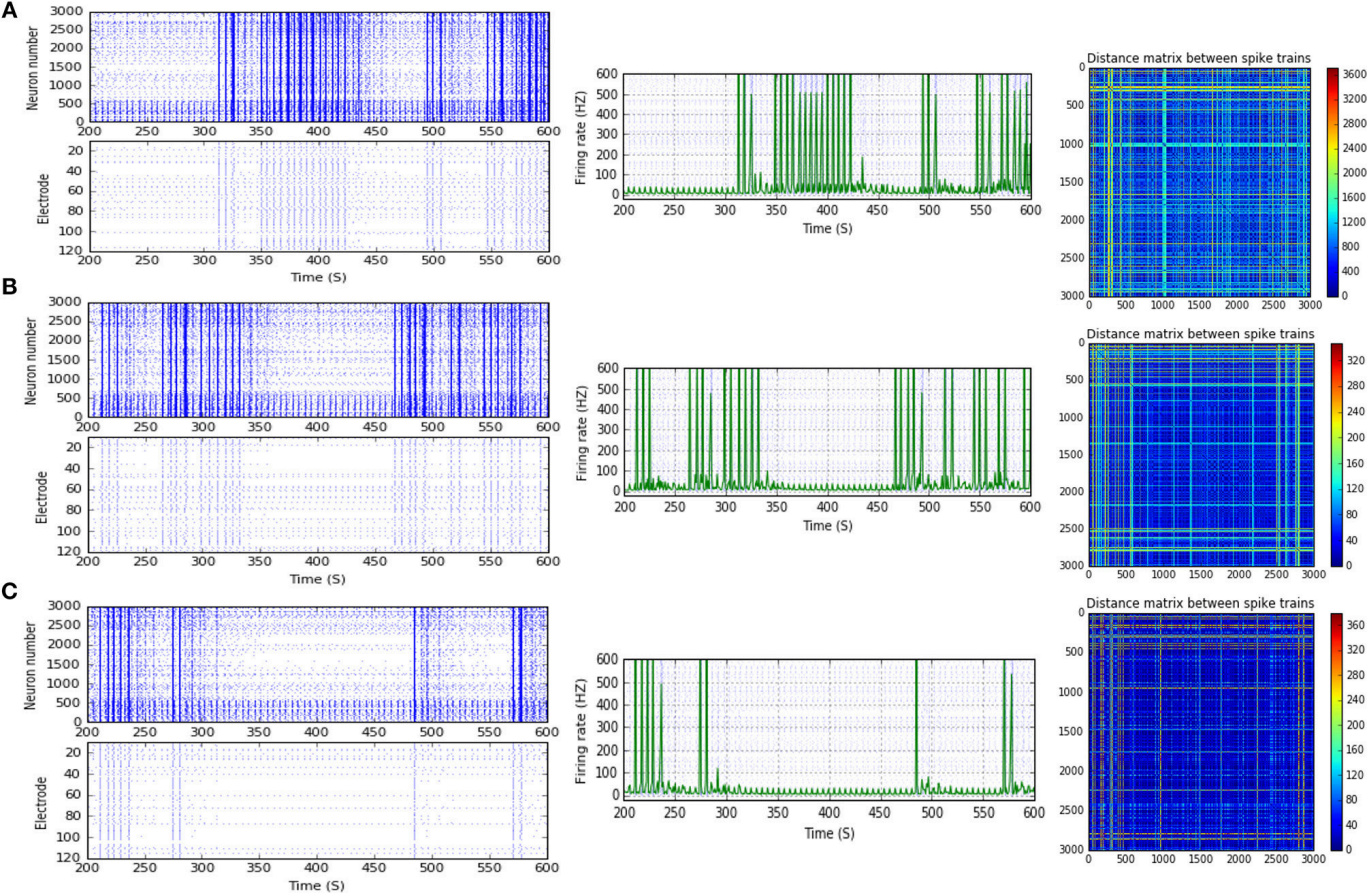
Selected elicited firing response activity in the immature network scenario (B-2) before and after implementing STF1 and STF2 (when IF = 30 Hz: in the range of beta). **(A)** refers to the condition without implementing dynamical synapses (control) while **(B,C)** refer to that after implementing facilitating synapses (STF1 and STF2, respectively). The first two figures of each panel refer to monitoring the pattern of firing activity in time windows from 200 to 600 seconds(s). Heat maps of each panel represent modulation changes in spike distance matrix of the network scenario (B-2) before and after implementing STF1 and STF2. The simulation time for heat maps of the spike distance is equal to 2,000 s.

Scenario (B-2) reveals a reduction of ~30.2%, from 25.8 to 18 Hz, in response to STD1 (when IF = 5 Hz: in the range of theta; Figures 3C, 4A,B). It also expresses a bigger reduction of about 67.9%, from 53.0 to 17.0 Hz, after implementing STF1 (when IF = 10 Hz: in the range of alpha; Figures 3C, 5A,B). Similarly, STF1 and STF2 (when IF = 15 Hz: in the range of beta) trigger a higher level of reduction in produced MSF (~61.7%, from 47.0 to 18.0 Hz and 48.9%, from 47.0 to 24.0 Hz, respectively; Figures 3C, 6). Additionally, implementing STF1 (when IF = 20 Hz: in the range of beta) to (B-2) induces a considerable reduction in MSF amount (~22.5%, from 54.0 to 35.8 Hz; Figures 3C, 7). However, the same scenario (B-2) revealed a reduction of about 33.7% (from 85.0 to 49.0 Hz) and 48.5% (from 85.0 to 43.8 Hz), after implementing STF1 and STF2, respectively (when IF = 30 Hz: in the range of beta; Figure 8).

The last three reduction outcomes showed a similar trend, which might imply a dynamical shift in development. This amount of MSF was experimentally reported to be in the range of 54.27 ± 7.22 Hz at this stage (Table 1; Baltz et al., 2010).

### Dynamical Synapses Sustain the Survival of Network Activity by Extracting the Extra Amount of Noise

STP (dynamical synapses) strongly modulated the firing activity of several network scenarios (B-2), (B-3), and (B-4) by inducing a remarkable reduction in the amount of MSF. This might propose a dynamical developmental shift comparable to what was previously experimentally reported *in vitro* (MEAs model) by the end of week 2 (Table 1; Baltz et al., 2010). This amount of MSF was experimentally reported to be in the range of 54.27 ± 7.22 Hz at this stage (Table 1; Baltz et al., 2010).

In response to IF, scenario (B-2) in the range of beta (30 Hz), (B-3) [high gamma (80 Hz)], and (B-4) [low gamma (40 Hz)], express reduction in the amount of MSF after implementing STP. STF1 and STF2 (when IF = 30 Hz: in the range of beta) induce a considerable but critical reduction in MSF amount (B-2). These reductions are ~42.3% (from 85.0 to 49.0 Hz) and about 48.5% (from 85.0 to 43.8 Hz) after implementing STF1 and STF2, respectively (Figures 3C, 8). On the other hand, STD1 strongly induces a significant reduction in MSF amount in both network scenarios, namely (B-3) when IF = 80 Hz (in the range of high gamma) and (B-4) when IF = 40 Hz (in the range of low gamma). In response to STD1, (B-3) shows a reduction of about 88.6%, from 133.8 to 15.2 Hz (when IF = 80 Hz: in the range of high gamma) while (B-4) induces a reduction of about 45%, from 110.0 to 60.5 Hz (when IF = 40 Hz: in the range of low gamma). This reduction is observed after implementing STD2 (when IF = 80 Hz: in the range of high gamma; Figure 12C).

Consequently, these decreases indicate a crucial dynamical shift from higher firing activity, which exceeded the experimentally reported value of MSF in normal developmental conditions at this stage (Table 1; Baltz et al., 2010). Thus, this modulation suggests a dynamical survival, as seen in a reduction shift of firing rate activity. Such reductions could resemble what might be observed in MEAs in a transitional period of development between week 1 and 2 *in vitro*.

In certain situations, network scenarios (A-1), (A-2), (B-1), (B-2), (B-3), and (B-4) expressed modulation in their firing activity in response to dynamical synapses, even though STP were not able to induce a sufficient developmental shift to resemble what might be observed experimentally during early developmental stages (first and second week) *in vitro* (Table 1; Baltz et al., 2010). Then, dynamical synapses might lose their ability to sustain the survival of network activity when it exceeds what was experimentally reported in MEAs model during the change in GABAergic synaptic transmission [before (5–10 DIV) and/or during (11–17 DIV) the GABA_A_ shift] (Table 1; Baltz et al., 2010).

In the previous examples, the induced modulation effects refer to two distinct situations. In the first situation, STP-triggered by all selected ranges of IF, in both network scenarios (A-1) and (A-2) does not succeed in expressing the amount of MSF similar to what was reported experimentally in MEAs during week 1 and 2 *in vitro* (Table 1; Baltz et al., 2010).

In the second situation, STP in response to specific IF in (B-1), (B-2), (B-3), and (B-4) does not express a similar amount of MSF compared to what could be recorded from MEAs during early stages of development (Table 1; Baltz et al., 2010).

After implementing STF2 (when IF = 20 Hz: in the range of beta), (B-1) shows about 20.3% increase, from 65.0 to 78.2 Hz (Figure 3A). It also expresses a 27.6% reduction, from 90.5 to 65.5 Hz, in response to STF2 (when IF = 45 Hz: in the range of low gamma) and a 47.6% reduction from 139.0 to 72.8 Hz, in response to STD1 (when IF = 80 Hz: in the range of high gamma; Figure 3A). Thus, despite the presence of STP modulation effects, it was not enough to adequately extort overflow of the firing activity. This observation indicates that it does not fall within the range of the experimentally reported MSF of MEAs model during the first and second week *in vitro* (Table 1; Baltz et al., 2010), i.e., 7.86 ± 1.30 Hz [before (5–10 DIV)] and 54.27 ± 7.22 Hz [during (11–17 DIV)]. Nevertheless, certain exceptional cases are illustrating substantial strength of dynamical synapses in gaining suitability for the survival of network activity by provoking the required modulation. This modulation signature mimics the amount of MSF that might be produced in normal conditions *in vitro* (Table 1; Baltz et al., 2010). For example, implementing STD1 and STF1 (when IF = 20 Hz: in the range of beta) to (B-1) leads to a high reduction in the amount of MSF (~44.6%, from 65.0 to 36.0 Hz and 41.5% from 65.0 to 38.0 Hz, respectively; Figure 3A). This high reduction might refer to a dynamical shift resembling experimental reports *in vitro*, that is, the recorded amount of MSF in the transitional developmental stage before the second week, just before the beginning of the second week and early in week 2 (Table 1; Baltz et al., 2010). Besides, the same scenario (B-1) shows a reduction of ~36.3%, from 62.5 to 39.8 Hz, in response to STD2 (when IF = 25 Hz: in the range of beta; Figure 3A).

**Figure 9:**
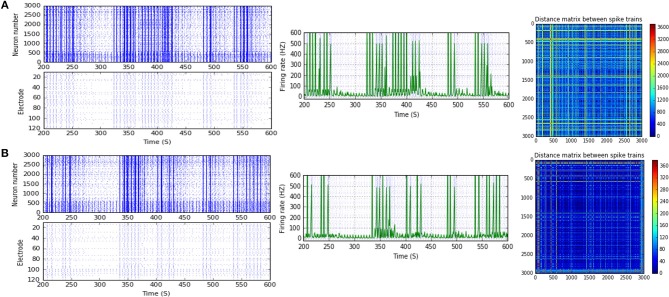
Selected elicited firing response activity in the immature network scenario (B-2) before and after implementing STF2 (when IF = 40 Hz: in the range of low gamma). **(A)** refers to the condition without implementing dynamical synapses (control) while **(B)** refers to that after implementing facilitating synapses (STF2). The first two figures of each panel refer to monitoring the pattern of firing activity in time windows from 200 to 600 seconds(s). Heat maps of each panel represent modulation changes in spike distance matrix of the network scenario (B-2) before and after implementing STF2. The simulation time for heat maps of the spike distance is equal to 2,000 s.

**Figure 10:**
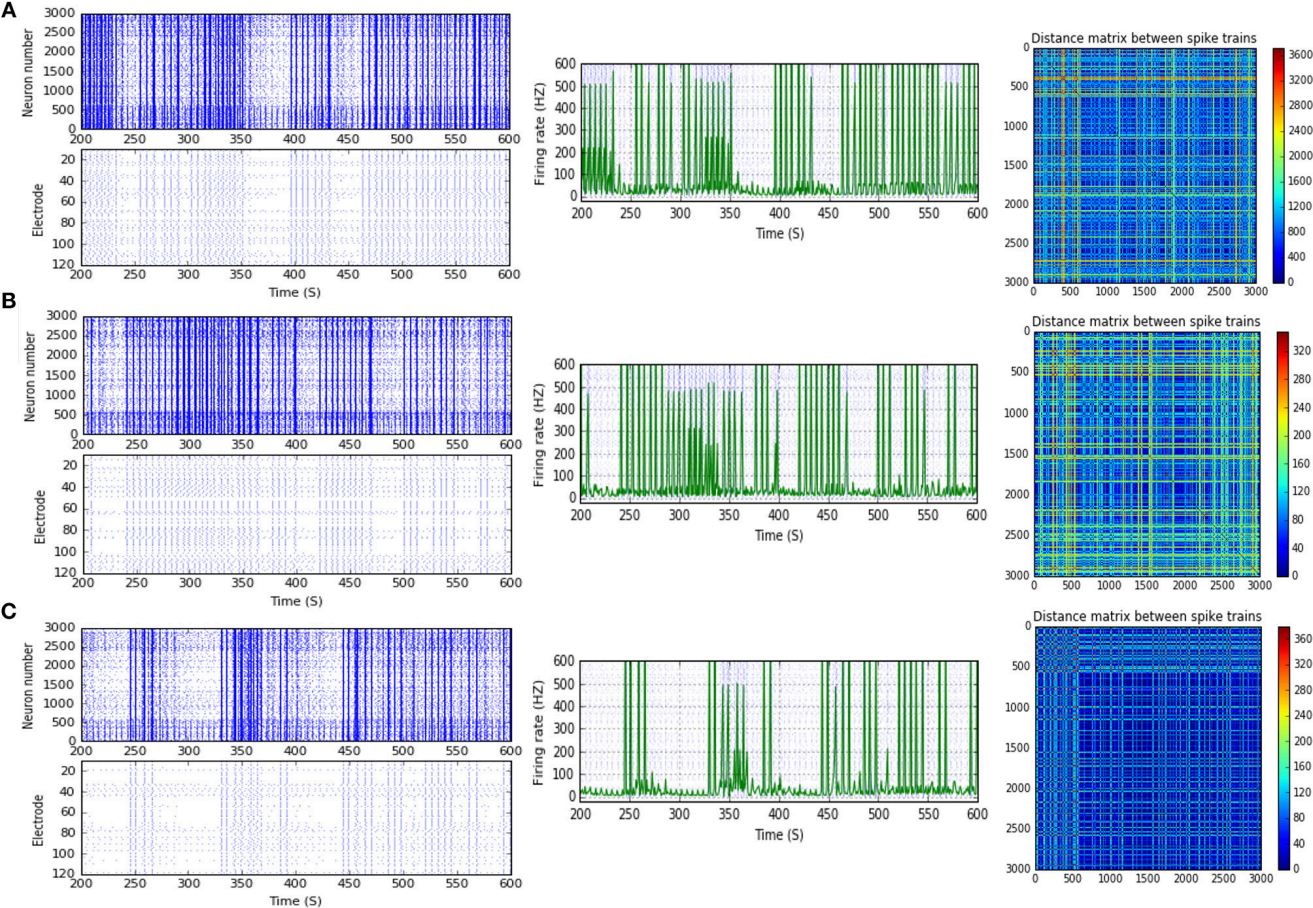
Selected elicited firing response activity in the immature network scenario (B-2) before and after implementing STF1 and STF2 (when IF = 50 Hz: in the range of low gamma). **(A)** refers to the condition without implementing dynamical synapses (control) while **(B,C)** refer to that after implementing facilitating synapses (STF1 and STF2, respectively). The first two figures of each panel refer to monitoring the pattern of firing activity in time windows from 200 to 600 seconds(s). Heat maps of each panel represent modulation changes in spike distance matrix of the network scenario (B-2) before and after implementing STF1 and STF2. The simulation time for heat maps of the spike distance is equal to 2,000 s.

**Figure 11:**
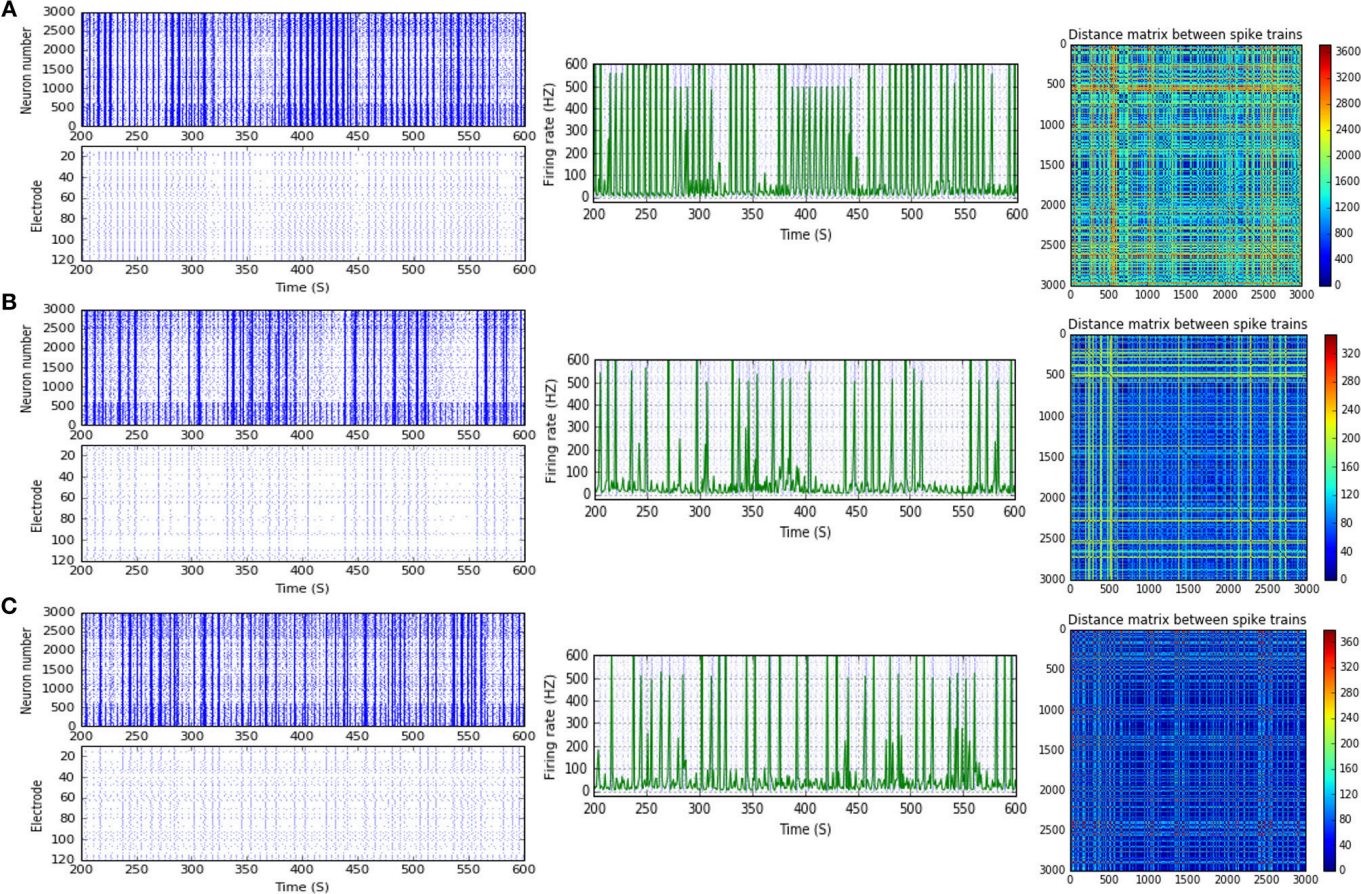
Selected elicited firing response activity in the immature network scenario (B-2) before and after implementing STD1 and STD2 (when IF = 100 Hz: in the range of high gamma). **(A)** refers to the condition without implementing dynamical synapses (control) while **(B,C)** refer to that after implementing depressing synapses (STD1 and STD2, respectively). The first two figures of each panel refer to monitoring the pattern of firing activity in time windows from 200 to 600 seconds(s). Heat maps of each panel represent modulation changes in spike distance matrix of the network scenario (B-2) before and after implementing STD1 and STD2. The simulation time for heat maps of the spike distance is equal to 2,000 s.

Moreover, (B-2) shows a considerable reduction in MSF amount, about 40.3% (from 112.0 to 66.8 Hz) in the presence of STF2 (when IF = 40 Hz: in the range of low gamma; Figures 3C, 9). Likewise, it expresses a slight reduction in MSF amount, about 4.6% (from 86.0 to 82.0 Hz) and 11.6% (from 86.0 to 76.0 Hz) in the presence of STF1 and STF2 (when IF = 50 Hz: in the range of low gamma), respectively (Figures 3C, 10). In contrast, STD2 (when IF = 100 Hz) induces a significant reduction (roughly about 41. 4%, from 152.7 to 89.5 Hz; Figures 3C, 11A,C). Nevertheless, the modulation effects in both situations are different from the experimentally reported values of MSF during the early stages of development *in vitro* (Table 1; Baltz et al., 2010).

**Figure 12:**
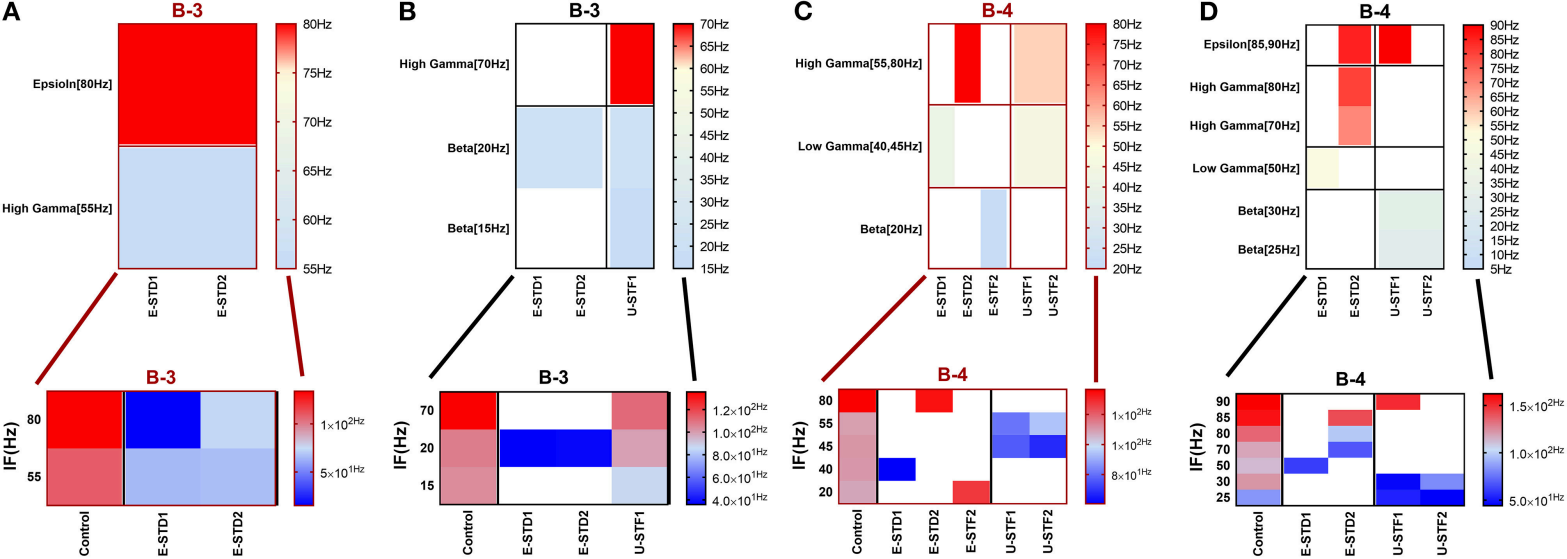
Heat maps of selected elicited responses in each network scenario (B-3) and (B-4), in both immature and mature conditions, respectively, before and after implementing dynamical synapses. **(A,C)** refer to immature conditions of network scenarios (B-3 and B-4) while **(B,D)** relate to mature conditions of network scenarios (B-3 and B-4). The upper graphs refer to particular Poisson input frequencies [IF (Hz)] that triggered a substantial modulation effect, measured in Hz before and after STP in these networks (Lower graphs). White blocks refer to excluded values due to statistical variations in probability density function (PDF). Control refers to the condition without STP implementation (dynamical synapses); STD1 and STD2 refer to two types of depressing synapses while STF1 and STF2 refer to facilitating synapses. E-STD1, E-STD1, E-STF1, and E-STF2 relate to the expected modulation effect induced by dynamical synapses, i.e., reduction of firing rate activities in response to depressing synapses (STD1 and STD2) while increasing the firing rate activity in response to facilitating synapses (STF1 and STF2). On the other hand, U-STD1, U-STD1, U-STF1, and U-STF2 relate to the expected modulation effect induced by dynamical synapses, i.e., an increase of firing rate activities in response to depressing synapses (STD1 and STD2), while reduction in the firing rate activity in response to facilitating synapses (STF1 and STF2), see also Tables 5, 6.

For (B-3), STD1 and STD2 (when IF = 55 Hz: in the range of high gamma) induce a remarkable decrease in MSF (about 40%, from 108.2 to 64.9 Hz and about 38.5%, from 108.2 to 66.5 Hz). Furthermore, STD2 (when IF = 80 Hz: in the range of high gamma) expresses a remarkable reduction in MSF, about 45.6% (from 133.8 to 72.8 Hz). Despite observing a reduction in firing activity, it is beyond the normal range of MSF during early stages of development, which was experimentally reported (Table 1; Baltz et al., 2010). Lastly, scenario (B-4) reveals certain conditions, through which STD2, STF1, and STF2 do not trigger the required modulation effects that could resemble what was experimentally reported during early stages of development, *in vitro* (Table 1; Baltz et al., 2010). For instance, (B-4) expresses a considerable increase in MSF amount, about 17.9% (from 107.7 to 127.0 Hz) in the presence of STF2 (when IF = 20 Hz: in the range of beta; Figure 12C). Moreover, it induces a remarkable reduction in MSF amount, roughly 31.2% (from 110.5 to 76.0 Hz) and 39.2% (from 110.5 to 67.2 Hz), in the presence of STF1 and STF2 (when IF = 45 Hz: in the range of low gamma), respectively (Figure 12C). Similarly, (B-4) expresses a considerable reduction in the amount of MSF, about 25.7% (from 109.1 to 81.0 Hz) and 13.4% (from 109.1 to 94.5 Hz) in the presence of STF1 and STF2 (when IF = 55 Hz: in the range of high gamma), respectively (Figure 12C). On the other hand, (B-4) triggers a slight reduction in the amount of MSF, about 2.2% (from 136.5 to 133.5 Hz) in the presence of STD2 (when IF = 80 Hz: in the range of high gamma; Figure 12C).

### A Significant Influence of STP on the Two Physiological States of GABA_A_

The differential significant modulation effects of each class of dynamical synapses (STD1, STD2, STF1 and STF2) triggered by IF in eliciting firing rate activity in each network scenario (immature and mature conditions), namely (A-1), (A-2), (B-1), (B-2), (B-3), and (B-4) is illustrated in the following section (Tables 5, 6).

### Immature Network Scenarios

Analyzed with a two-way ANOVA, the elicited modulated effects, induced by IF (Hz) and dynamical synapses (STP) in an immature condition within its network scenarios (i.e., each network scenario related to immature condition) are shown in Table 5 and summarized as follows:

1. STD1 revealed significant effects [*P* = 0.0056 (A-1), *P* = 0.0141(A-2), *P* < 0.0001(B-1), *P* = 0.0138 (B-3), and *P* = 0.0010 (B-4)], while IF (Hz) had a significant effect on (B-3) (*P* = 0.0420). As to depressing synapses (STD1), which expressed reduction in the firing rate activity by STD1 (E-STD1), IF (Hz) induced significant effects [*P* = 0.0048 (B-1) and *P* = 0.0087 (B-2)], while E-STD1 expressed significant effects [*P* < 0.0001(B-1), *P* = 0.0003 (B-2), and *P* < 0.0001 (B-4)]. On the other hand, the unexpected elicited modulation effect induced by depressing synapses (STD1) expressed an increase in the firing rate activity by STD1 (U-STD1). IF (Hz) induced a significant effect [*P* = 0.0001 (B-3)] while U-STD1 expressed significant effects [*P* = 0.0361 (A-2), *P* = 0.0084 (B-2), and *P* = 0.0027 (B-3)].
2. STD2 expressed significant effects [*P* = 0.0360 (A-1), *P* = 0.0143 (A-2), *P* = 0.0143 (B-1), *P* = 0.0328 (B-3), and *P* = 0.0008 (B-4)], while IF (Hz) had a significant effect on (B-3) (*P* = 0.0419). As for the expected elicited modulation effect induced by depressing synapses (STD2), which expressed reduction in the firing rate activity by STD2 (E-STD2), IF (Hz) induced significant effects [*P* = 0.0011 (B-2)], while E-STD2 expressed significant effects [*P* = 0.0034(A-1), *P* = 0.0001(A-2), *P* < 0.0001(B-1), *P* = 0.0002(B-2), and *P* < 0.0001(B-4)]. Concerning the unexpected elicited modulation effect induced by depressing synapses (STD2), which expressed an increase in the firing rate activity by STD2 (U-STD2), IF (Hz) did not induce any significant effects at any network scenario, however, U-STD2 expressed significant effects [*P* = 0.0379(A-2), *P* = 0.0185(B-1), *P* = 0.0254(B-2), *P* = 0.0216(B-3), and *P* = 0.0342(B4)].
3. STF1 expressed significant effects [*P* = 0.0047 (B-1) and *P* = 0.0127 (B-4)], while IF (Hz) had significant effects on (B-1) and (B-2) (*P* = 0.0268 and *P* < 0.0001, respectively). As to the expected elicited modulation effect induced by facilitating synapses (STF1), which expressed an increase in the firing rate activity by STF1 (E-STF1), IF (Hz) induced a significant effect [*P* = 0.0179 (A-2)], while E-STF1 expressed significant effects [*P* = 0.0179 (A-2) and *P* = 0.0055 (B-3)]. Concerning the unexpected elicited modulation effect induced by facilitating synapses (STF1), which expressed a reduction in the firing rate activity by STF1 (U-STF1), IF (Hz) induced significant effects [*P* = 0.0174 (A-1), *P* = 0.0135 (B-1), *P* = 0.0211 (B-2), and *P* = 0.0023 (B-3)], while U-STF1 expressed significant effects [*P* = 0.0238 (A-2), *P* = 0.0008 (B-1), *P* = 0.0156 (B-2), and *P* = 0.0006 (B-3)].
4. STF2 expressed significant effects [*P* = 0.0464 (B-1), *P* = 0.0057(B-2), and *P* = 0.0031(B-3)], while IF (Hz) had significant effects on (B-1), (B-2), and (B-3) (*P* = 0.0273, *P* = 0.0040, and *P* = 0.0031, respectively). As to the expected elicited modulation effect induced by facilitating synapses (STF2) [which expressed an increase in the firing rate activity by STF2 (E-STF2)] IF (Hz) induced significant effects [*P* = 0.0234(A-1), *P* = 0.0296(A-2), *P* = 0.0076(B-1), *P* = 0.0027(B-3), and *P* = 0.0036(B-4)] as well as E-STF2 that expressed significant effects [*P* = 0.0233(A-1), *P* = 0.0165(B-1), *P* = 0.0024(B-2), *P* = 0.0212(B-3), and *P* = 0.0203(B-4)]. Regarding the unexpected elicited modulation effect induced by facilitating synapses (STF2), which expressed a reduction in the firing rate activity by STF2 (U-STF2), IF (Hz) induced significant effects [*P* = 0.0036 (B-1), *P* = 0.0086 (B-2), and *P* = 0.0036 (B-3)], while U-STF2 expressed significant effects [*P* = 0.0048 (A-2), *P* < 0.0001(B-1), *P* = 0.0139 (B-2), and *P* = 0.0030 (B-3)].

### Mature Network Scenarios

The results of the two-way ANOVA of the elicited modulated effects induced by IF (Hz) and dynamical synapses (STP) in the mature condition, within its network scenarios are illustrated in Table 6 and can be summarized as follows:

1. STD1 expressed significant effects [*P* = 0.0438 (A-2), *P* = 0.0001(B-1), *P* = 0.0025 (B-2), *P* = 0.0144 (B-3), and *P* = 0.0050 (B-4)], while IF (Hz) had significant effects on (A-1) and (B-1) (*P* = 0.0286 and *P* = 0.0126). As to the expected elicited modulation effect induced by depressing synapses (STD1), which expressed reduction in the firing rate activity by STD1 (E-STD1), IF (Hz) induced significant effects [*P* < 0.0001(A-1), *P* = 0.0083 (A-2), *P* = 0.0043(B-1), *P* = 0.0014(B-3), and *P* = 0.0301(B-4)], while E-STD1 expressed significant effects [*P* = 0.0004 (A-1), *P* < 0.0001(A-2), *P* < 0.0001(B-1), *P* < 0.0001(B-2), *P* < 0.0001(B-3), and *P* < 0.0001(B-4)]. On the other hand, the unexpected elicited modulation effect induced by depressing synapses (STD1), which expressed an increase in the firing rate activity by STD1 (U-STD1), IF (Hz) induced significant effects [*P* = 0.0139 (B-1) and *P* = 0.0415 (B-4)], while U-STD1 expressed a significant effect [*P* = 0.0286 (B-4)].
2. STD2 expressed significant effects [*P* = 0.0002 (A-1), *P* = 0.0004 (B-1), *P* < 0.0001 (B-2), *P* = 0.0101 (B-3), and *P* = 0.0027 (B-4)], while IF (Hz) had significant effects on (A-1), (B-1), (B-2), (B-3), and (B-4) (*P* < 0.0001, *P* = 0.0237, *P* = 0.0439, *P* = 0.0490, and *P* = 0.0064, respectively). As to the expected elicited modulation effect induced by depressing synapses (STD2), which expressed reduction in the firing rate activity by STD2 (E-STD2), IF (Hz) induced significant effects [*P* < 0.0001(A-1), *P* = 0.0008(A-2), *P* = 0.0109 (B-1), *P* = 0.0439 (B-2), and *P* = 0.0011(B-4)], while E-STD2 expressed significant effects [*P* < 0.0001 (A-1), *P* = 0.0004 (A-2), *P* < 0.0001 for (B-1), (B-2), (B-3), and (B-4)]. Regarding the unexpected elicited modulation effect induced by depressing synapses (STD2), which expressed an increase in the firing rate activity by STD2 (U-STD2), IF (Hz) induced significant effects on (A-1), (B-1), and (B-3) (*P* = 0.0035, *P* = 0.0022, and *P* = 0.0070, respectively), however, U-STD2 expressed significant effects [*P* = 0.0330 (A-1) and *P* = 0.0323 (B-1)].
3. STF1 expressed significant effects [*P* = 0.0042 (B-1), *P* = 0.0275 (B-2), *P* = 0.0081(B-3) and *P* = 0.0009 (B4)], while IF (Hz) had significant effects on (A-2), (B-1), (B-2), (B-3), and (B-4) (*P* = 0.0084, *P* = 0.0002, *P* = 0.0337, *P* = 0.0035, and *P* = 0.0049, respectively). As to the expected elicited modulation effect induced by facilitating synapses (STF1), which expressed an increase in the firing rate activity by STF1 (E-STF1), IF (Hz) induced significant effects [*P* = 0.0205 (A-1), *P* = 0.0364 (B-1), and *P* = 0.0383 (B-2)], while E-STF1 expressed significant effects [*P* = 0.0170 (A-1) and *P* = 0.0275 (B-1)]. Concerning the unexpected elicited modulation effect induced by facilitating synapses (STF1), which expressed a reduction in the firing rate activity by STF1 (U-STF1), IF (Hz) induced significant effects [*P* = 0.0371(A-1), *P* = 0.0043 (A-2), *P* < 0.0001(B-1), *P* = 0.0196 (B-2), *P* = 0.0005 (B-3), and *P* = 0.0008 (B-4)], while U-STF1 expressed significant effects [*P* = 0.0048 (A-1), *P* = 0.0018 (A-2), *P* < 0.0001(B-1), *P* = 0.0023 (B-2), *P* < 0.0001(B-3), and *P* < 0.0001(B-4)].
4. STF2 expressed significant effects [*P* = 0.0011(B-1) and *P* = 0.0059 (B-3)], while IF (Hz) had significant effects on (B-1), (B-3), and (B-4) (*P* = 0.0002, *P* = 0.0015, and *P* = 0.0402, respectively). As for the expected elicited modulation effect induced by facilitating synapses (STF2), which expressed an increase in the firing rate activity by STF2 (E-STF2), IF (Hz) induced significant effects [*P* = 0.0028 (B-1) and *P* = 0.0003 (B-3)], while E-STF2 expressed significant effects [*P* = 0.0285 (A-1), *P* = 0.0255 (A-2), *P* = 0.0069 (B-2), and *P* = 0.0156 (B-4)]. Regarding the unexpected elicited modulation effect induced by facilitating synapses (STF2), which expressed a reduction in the firing rate activity by STF2 (U-STF2), IF (Hz) induced significant effects [*P* = 0.0111(A-2), *P* < 0.0001(B-1), *P* = 0.0031(B-3), and *P* = 0.0110 (B-4)], while U-STF2 expressed significant effects [*P* = 0.0008 (A-1), *P* < 0.0001(A-2), *P* < 0.0001(B-1), *P* = 0.0030 (B2), *P* = 0.0002 (B-3), and *P* = 0.0006 (B-4)].

## Discussion

Investigations do not reveal a consensus on the type of non-linearities in synapses at different maturational stages of the neocortex (Baltz et al., 2010). Therefore, we present herein a simulation study under various network scenarios, based on incorporating particular percentages of a local density of dendritic arborization (i.e., lateral connectivity between neurons) and a lateral spread length between neighboring neurons (i.e., the nearby local connectivity). Our results provide a prediction of how several neural network scenarios, including GABA_A_ switch, lateral and local connectivity, and dynamical synapses, interact during cortical development.

### Monitoring Firing Responses

#### Through Measuring MSF

Before implementing STP, we observed a certain level of variability in the produced amount of MSF between network scenarios of mature and immature condition. This validates the independent nature of each scenario. Furthermore, the increment in the amount of MSF was associated with the increase in the values of IF (Hz). Nevertheless, there were notable differences between immature and mature conditions. For instance, before implementing the dynamical synapses, there was no significant impact of IF on eliciting firing responses among all the network scenarios of the immature condition. In contrast, IF expressed significant effects on the firing responses among all the network scenarios of the mature condition. This observation might reflect the relation between the selectivity of IF and the maturation nature of the network based on the physiology of the GABAergic influence. However, some exceptional situations expressed certain degrees of fluctuation, in particular, after implementing dynamical synapses. These fluctuations were distinctively shown in each scenario, thus indicating their dynamical nature, as well as for immature and mature conditions, reflecting the impact of the GABAergic signature. Therefore, we argue that higher IF values (in ranges of high gamma and epsilon) do not often lead to higher firing responses in both conditions (immature and mature), i.e., the inducing effect of such values on the immature network scenarios lead to higher firing responses in comparison with mature network scenarios (Khalil et al., 2017b). Consequently, IF (Hz) is not the exclusive driving factor, as there are additional fundamental elements, such as the proportion of lateral and local connectivity, and more importantly, the physiological state of GABA_A_.

After implementing dynamical synapses (STP), all network scenarios—of both conditions—expressed remarkable differences in the produced amount of MSF.

In general, the effects of depressing synapses support the findings of Loebel and Tsodyks (2002). These effects have been thoroughly explained in many studies (Tsodyks and Markram, 1997; Tsodyks et al., 1998; Loebel and Tsodyks, 2002). Facilitating synapses showed high dynamical variability, i.e., reduction and increase in MSF amount, in both conditions (immature and mature). Nevertheless, the slight increment in the amount of MSF in response to STF is in line with the study of Barak and Tsodyks (2007). They proposed that facilitating synapses (STF) serve to optimize information transfer for high firing rate activity due to the rise in synaptic strength. Hence, one can argue that during later stages of development (weeks 3 and 4 *in vitro*), dynamical facilitating synapses can develop their synaptic strength within a certain limit. This argument is further indicated through varying synaptic non-linearity parameters that influence the structure of each network scenario, which explains the observed reduction in MSF across scenarios. Together, these effects may temporally synchronize with the physiological maturation of GABA_A_ during early stages of cortical development (weeks 1 and 2 *in vitro*).

To conclude, dynamical synapses expressed two contrasting modulation effects among network scenarios of both conditions (immature and mature). One effect indicated its substantial contribution in producing a remarkable and adequate reduction in the amount of MSF to resemble what is biologically observed in MEA models during these stages of development. Another revealed the failure of STP to maintain the survival of network activity when MSF was high, exceeding what might be experimentally observed in MEAs during the first 2 weeks (immature network scenarios) and the third and fourth week (mature network scenarios) *in vitro*. Therefore, in certain circumstances, dynamical synapses could express a capacity to influence the network survival. Thus, it would remain in the normal developmental range of what was seen in the *in vitro* model in various stages of development by inducing a significant modulator effect. These effects would be capable of producing the normal amount of MSF. We also demonstrated how STP could modulate the amount of MSF, which implies a developmental shift to earlier developmental stages *in vitro*, either within the first 2 weeks, the third and fourth week or slightly earlier. Accordingly, abundant supplies of STP signaling [triggered by IF (Hz)] might resemble an intrinsic driving factor for shaping the pattern of firing activity during different developmental stages *in vitro*.

#### Through Measuring Firing Rate Activity (Hz)

Each STP type induced two contrasting modulation effects with variation from one network scenario to another in mature and immature conditions. Accordingly, it was imperative to separate these responses into two parts: predicted and unpredicted. The former refers to the success of depressing synapses (i.e., STD) in inducing a significant reduction in neural firing activity. This reduction is in agreement with the study of Barak and Tsodyks (2007). In contrast, the latter points to the opposite effect of STD, which is not in line with the majority of short-term plasticity studies. Nevertheless, states of predicted modulation effects were lower in the case of depressing synapses (i.e., modulation through reduction) and higher in the case of facilitating synapses (i.e., modulation through increasing firing activity in immature condition) and vice versa (i.e., mature condition).

On the other hand, unpredicted modulation effects were higher in the case of STD (i.e., modulation through increasing firing activity) and lower in the case of facilitating synapses (i.e., modulation through a reduction in immature condition) and vice versa. Our finding supports the suggestion of the diverse functions of STP components in synaptic computations (Deng and Klyachko, 2011; Blackman et al., 2013; Larsen and Sjöström, 2015). Then, there is a possibility of having developmental STP switch, which serves for a particular function.

According to Cheetham and Fox (2010), during the early development, there is a strong short-term synaptic depression of excitation, which is essential to avoid the uncontrolled excitation due to the partial maturation of the inhibition at this time. Nevertheless, in the mature condition, having “fully developed inhibition” may cancel the need for this strong depression, thus allowing excitatory synapses to express “a richer spectrum of short-term dynamics” (Cheetham and Fox, 2010). One of the appealing possibilities according to Blackman et al. (2013), is that the dynamics of several synapse types may mature in a differential manner. i.e., synapse types with similar STP may become dissimilar with age while those with different subclasses of synaptic dynamics (i.e., depressing synapses (STD1, STD2) and facilitating synapses (STF1 and STF2) in the immature condition may be similar until the neural network reaches the level of maturation. However, this possibility has not yet been extensively explored. Therefore, one can claim that STP expresses a differential potential to influence the network activity, based on its membrane time constant, IF (Hz), the network structure, and the physiological state of GABA_A_.

### Dynamical Synapses and the Modulation of Neural Network Activity Through Synaptic Fine Tuning

After the implementation of dynamical synapses, we observed the following: (1) differential effects of IF in inducing the modulated responses, which is expressed in the noticeable changes in the firing rate activity, among network scenarios for both conditions (immature and mature networks). These differential effects refer to the significant effects of IF in expressing modulated firing activity after implementing; depressing synapses (STD1 and STD2) in immature condition, and facilitating synapses (STF1 and STF2) in mature condition. In contrast, there was a slight but not significant effect of IF in expressing modulated firing activity after implementing; depressing synapses (STD1 and STD2) in mature condition, and facilitating synapses (STF1 and STF2) in immature condition. (2) The modulation responses, which were elicited after the implementation of dynamical synapses, expressed differently in each network scenario for both conditions (immature and mature networks). Overall, there was a significant modulation effect in response to dynamical synapses (STP). However, the degree of significance varied based on the state of GABA_A_ reversal potential, which referred to the maturation of the neural networks. This observation confirms the crucial role of the physiological state of GABA_A_ in response to dynamical synapses. Thus, our findings reflect the crucial role of STP in modulating neural network activity by mediating several ranges of IF through two physiological states of GABA_A_. This finding is in line with fundamental studies on STP mechanisms (Tsodyks and Markram, 1997; Tsodyks et al., 1998; Loebel and Tsodyks, 2002) highlighting the essential role of STP in filtering signal propagation to sustain and maintain neural network activity.

Each class of STP showed two contrasting modulation effects not only with the variation in each network scenario but also from one condition to another, i.e., immature and mature network condition. Notably, there were differential effects of IF in inducing the modulated responses, expressed in noticeable changes in the firing rate activity after implementing dynamical synapses, among network scenarios for both conditions (immature and mature networks). Thus, IF induced significant effects in expressing modulated firing activity after implementing; (1) depressing synapses (STD1 and STD2) in immature condition, and (2) facilitating synapses (STF1 and STF2) in mature condition. In contrast, IF (Hz) did not reveal a significant effect in expressing modulated firing activity after implementing; depressing synapses (STD1 and STD2) in mature condition, and implementing facilitating synapses (STF1 and STF2) in immature condition. This observation indicates the crucial impact of IF in modulating the firing rate responses, and its relation to dynamical synapses and the GABAergic physiological state of maturation. Therefore, it was necessary to segregate these modulated responses (according to studies on STP) into two sections: Predicted and Unpredicted. The first modulation effect referred to the substantial contribution of depressing synapses in inducing a remarkable and sufficient reduction in the neural firing rate activity (Hz) while increment effect in case of facilitating synapses.

On the other hand, the contrasting effect of STF relates to unpredicted influence, which is not in agreement with the majority of STP plasticity studies. Noticeably, the number of predicted modulation effects induced by depressing synapses (STD) was lower in comparison with facilitating synapses (STF) in immature condition and vice versa in mature condition. Therefore, STP might express a capacity to influence network activity based on the physiological state of GABA_A_. Thus, reflecting the diverse functions of STP components in synaptic computations (Deng and Klyachko, 2011; Blackman et al., 2013; Larsen and Sjöström, 2015), then, there are potential functions and many mechanisms underlying “target-specific STP.” There are several examples of “STP specific to the target cell (Angulo et al., 1999; Reyes and Sakmann, 1999; Buchanan et al., 2012; Costa et al., 2013), and despite the existence of a few common principles, it is clear that not nearly enough is known about the why and the how.” Therefore, the specificity of STP should be explored elaborately, both theoretically and empirically (Blackman et al., 2013).

### Clinical Implications and Future Directions

STP is an essential element for evaluating firing rate activity (Hz) during the physiological development of GABA_A_ signaling. Consequently, STP might represent an indicator for the interplay between the action of physiological maturation of GABA_A_ signaling on one hand and the functional maturation of both the local and lateral connectivity between neurons on the other hand. Therefore, our study might have a potential biological relevance for more complex biological network scenarios. Thus, additional requisite puzzles, related to neocortical development from a biophysical perspective, could be approached using our SNN model. We consider our model as a robust predictive tool for further evaluation of the impact of functional and structural changes. These might be due to several factors, such as an excess or a deficit of IF or maladaptive modulation of dynamical synapses. A better understanding of GABAergic signaling in brain maturation and neuropsychiatric disorders can help develop novel treatment interventions. Ben-Ari et al. (2012) argued that since GABA depolarizes pathological neurons, then agents capable of reducing [Cl^−^]_i_ maybe of therapeutic value. Remarkably, it has been shown that Oxytocin-mediated reduction of [Cl^−^]_i_ exerts neuroprotective actions, reducing the severity of anoxic episodes (Tyzio et al., 2006) and also exerts analgesic actions, increasing the threshold of pain reactions (Mazzuca et al., 2011). Although several factors can mediate changes in [Cl^−^]_i_, they usually involve the chloride importer NKCC1 and the chloride exporter KCC2. Increased activity of NKCC1, as well as down-regulation of KCC2, have been observed in experimental and human epileptic neurons (Huberfeld et al., 2007; Ben-Ari et al., 2012). Although the GABA-acting antiepileptic drug phenobarbital (PB) is the drug of first choice to treat neonatal seizures (Bassan et al., 2008), in many cases, it fails to block or significantly reduce epileptic seizures (Guillet and Kwon, 2007). However, pioneering studies have found empirical evidence for the therapeutic role of diuretics that, by reducing [Cl^−^]_i_, facilitate the antiepileptic effects of PB and related drugs (Dzhala et al., 2008, 2010).

Moreover, further studies suggest alterations of GABAergic signaling in autism spectrum disorders (ASDs) (Zhang et al., 2010; Pizzarelli and Cherubini, 2011). Lemonnier and Ben-Ari (2010) investigated the effects of long-term administrations of the diuretic bumetanide on infants with ASD and found highly beneficial effects. Interestingly, Oxytocin has also been shown to transiently improve visual communication in adults with ASD (Andari et al., 2010). Thus, future studies will have to investigate the therapeutic effects of agents capable of reducing [Cl^−^]_i_ in the different subtypes of patients with ASDs showing different pathophysiological conditions (Krippl and Karim, 2009, 2011; for a review see Khalil et al., 2018). Recently, we suggested a multilayer neural network model for patients with ASDs including the mirror neuron system on a first layer and transforming this information to a higher layer network responsible for reasoning (Khalil et al., 2018). Future studies with ASD participants combining behavioral tasks with neuroimaging methods and pharmacological interventions as well as computational modeling can help validate and complement this suggested model and reveal the therapeutic role of GABAergic modulation in specific subtypes of ASDs and other neurological and psychiatric disorders.

## Author Contributions

RK and AM designed the study and performed the simulation and data analyses. RK and MM interpreted the data, wrote the manuscript and provided critical revisions. EK and AK provided clinical implications. AK and MM critically revised language and references for the final revisions.

### Conflict of Interest Statement

The authors declare that the research was conducted in the absence of any commercial or financial relationships that could be construed as a potential conflict of interest.

## Abbreviations

IF: Poisson input frequency
MEAs: multi-electrode arrays
SNN: spiking neural network
STD: short-term depression—depressing synapses
STF: short-term facilitation—facilitating synapses
STP: short-term synaptic plasticity—dynamical synapses.
