# Supplementary material for: Dynamic Communications Between GABAA Switch, Local Connectivity, and Synapses During Cortical Development: A Computational Study
Source: Front Cell Neurosci. 2018 Dec 17;12:468. doi: 10.3389/fncel.2018.00468 (PMC6304749; doi:10.3389/fncel.2018.00468)
Supplement: Supplementary file 1 [file Presentation_1.PDF]

# Supplementary Information

For the sake of selecting the most optimal profile of connectivity between inhibitory and excitatory neuronal populations, we focused on designing the connectivity structure of our network before implementing STP. Therefore, we followed a systematic procedure, in which we measured the sub threshold voltage of the neurons in response to changes in the connectivity profile between inhibitory and excitatory neuronal population (Figure S.1). Then, we monitored the firing activity for selecting the "best-fit "condition for the connectivity profile at an mV level (Figure S.2). Also, we provide supplementary information about selected Poisson input frequency (IF) in triggering modulation effect after implementing depressing and facilitating dynamical synapses across all network scenarios of both conditions: immature and mature networks(Figures; S.3 and S.4).Also, we illustrate represent the initial heat maps of the produced firing rate activity for each network scenarios; (A-1), (A-2), (B-1), (B-2), (B-3) and (B-4) respectively before and after implementing dynamical synapses; STD1, STD2, STF1 and STF2(Figures; S.5, S.6, S.7, S.8, S.9 and S.10).

# Supplementary Figures

**Figure S.1**

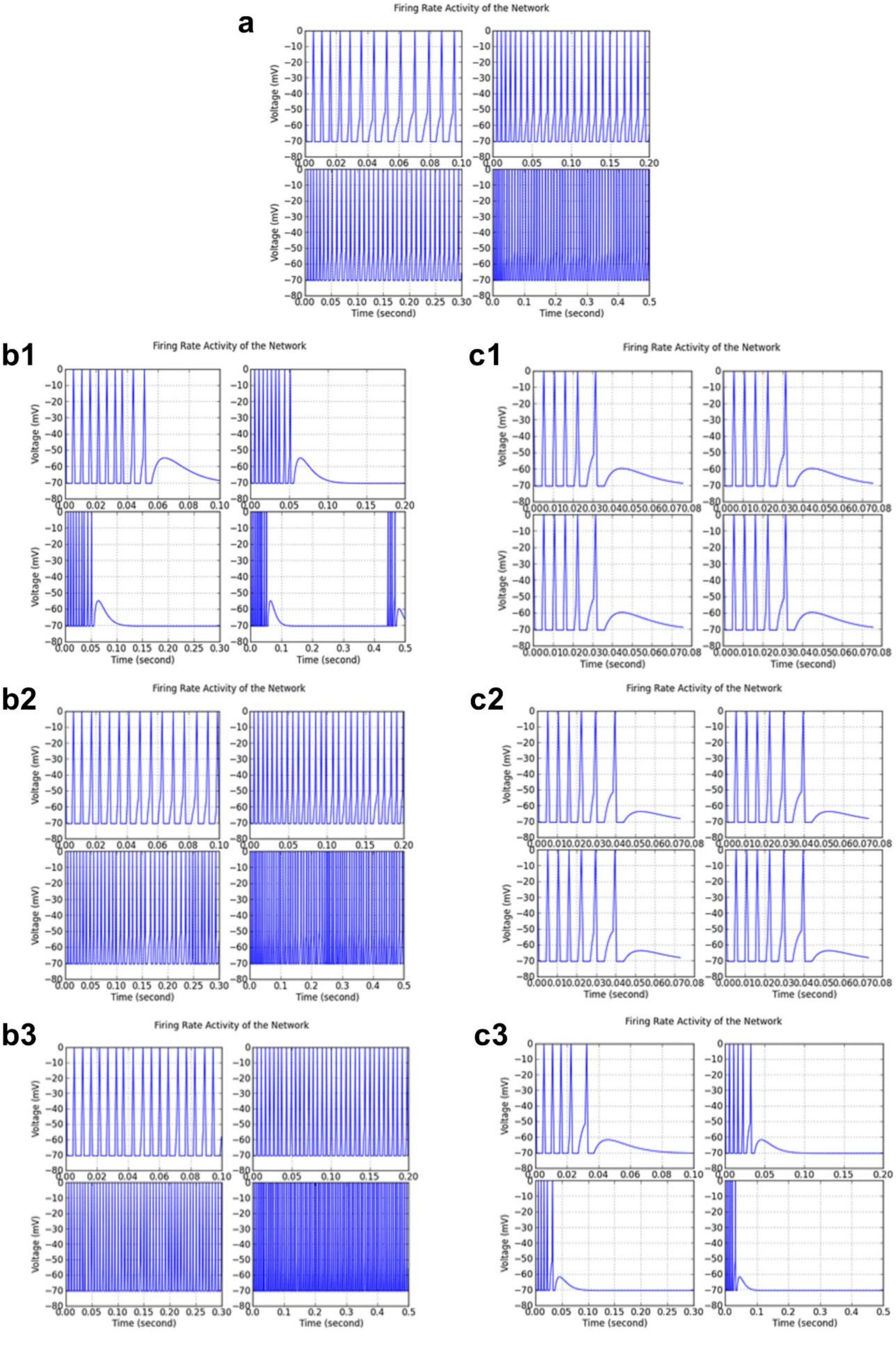
a
Firing Rate Activity of the Network
b1
Firing Rate Activity of the Network
c1
Firing Rate Activity of the Network
b2
Firing Rate Activity of the Network
c2
Firing Rate Activity of the Network
b3
Firing Rate Activity of the Network
c3
Firing Rate Activity of the Network
Voltage (mV)
Time (second)

**Figure S.2**

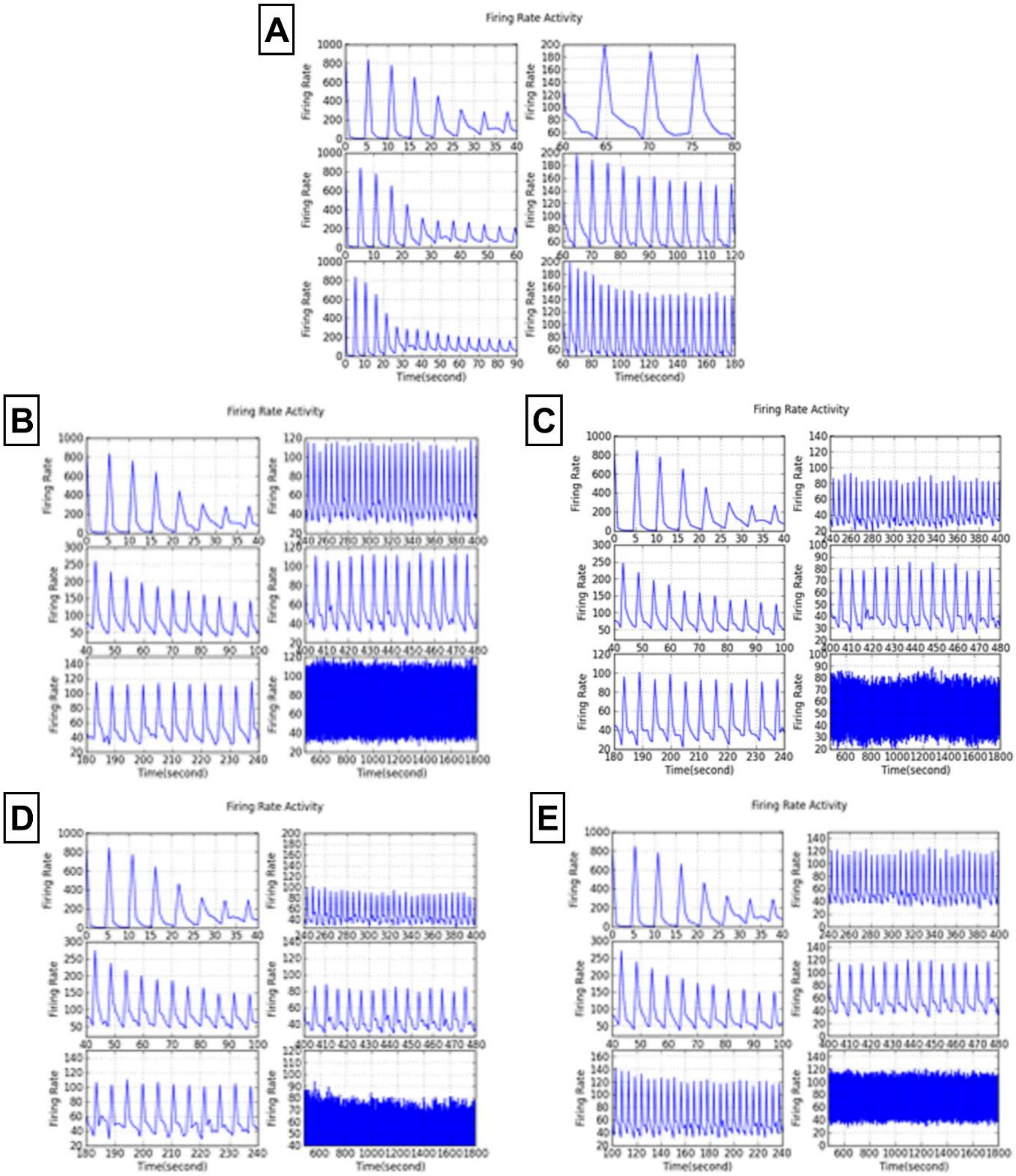
A
Firing Rate Activity
Firing Rate
Time(second)
B
Firing Rate Activity
Firing Rate
Time(second)
C
Firing Rate Activity
Firing Rate
Time(second)
D
Firing Rate Activity
Firing Rate
Time(second)
E
Firing Rate Activity
Firing Rate
Time(second)

**Figure S.3**

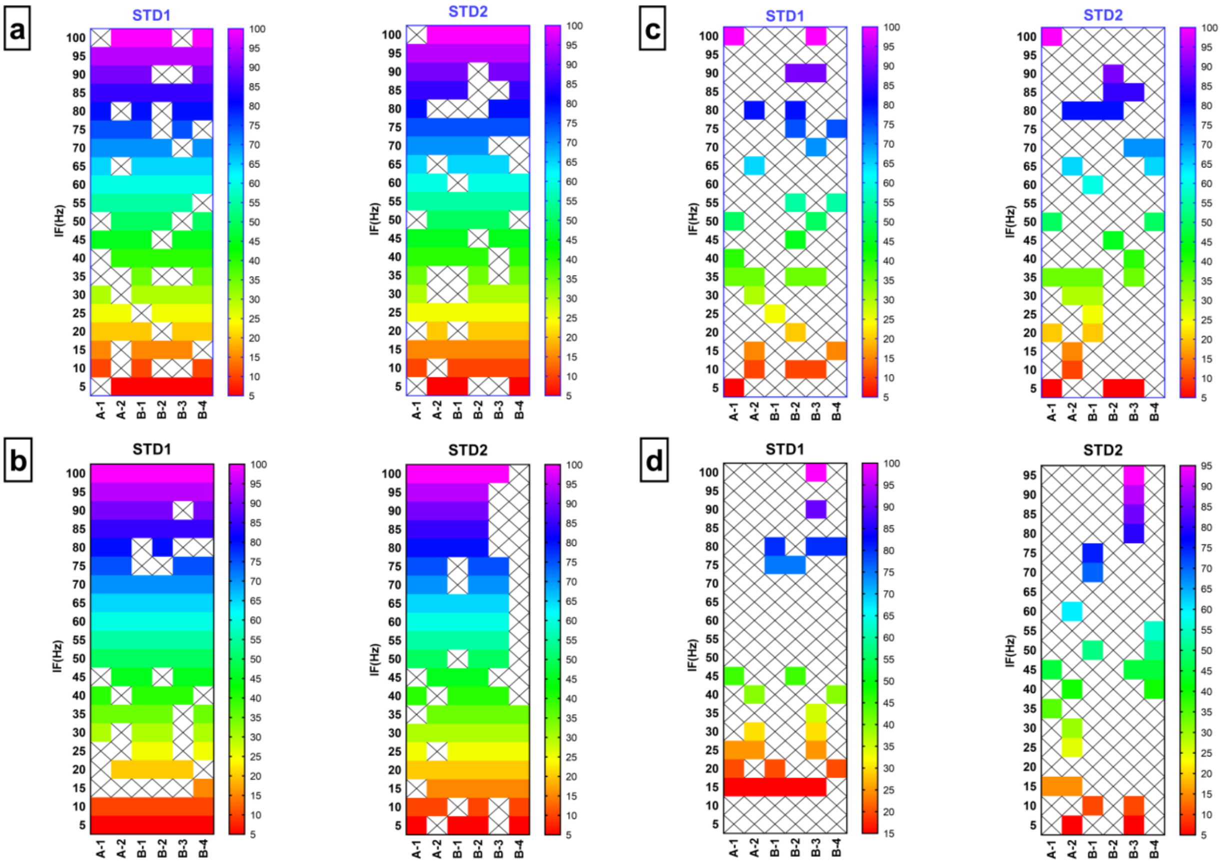
a
STD1
STD2
b
STD1
STD2
c
STD1
STD2
d
STD1
STD2
IF(Hz)
A-1
A-2
B-1
B-2
B-3
B-4

**Figure S.4**

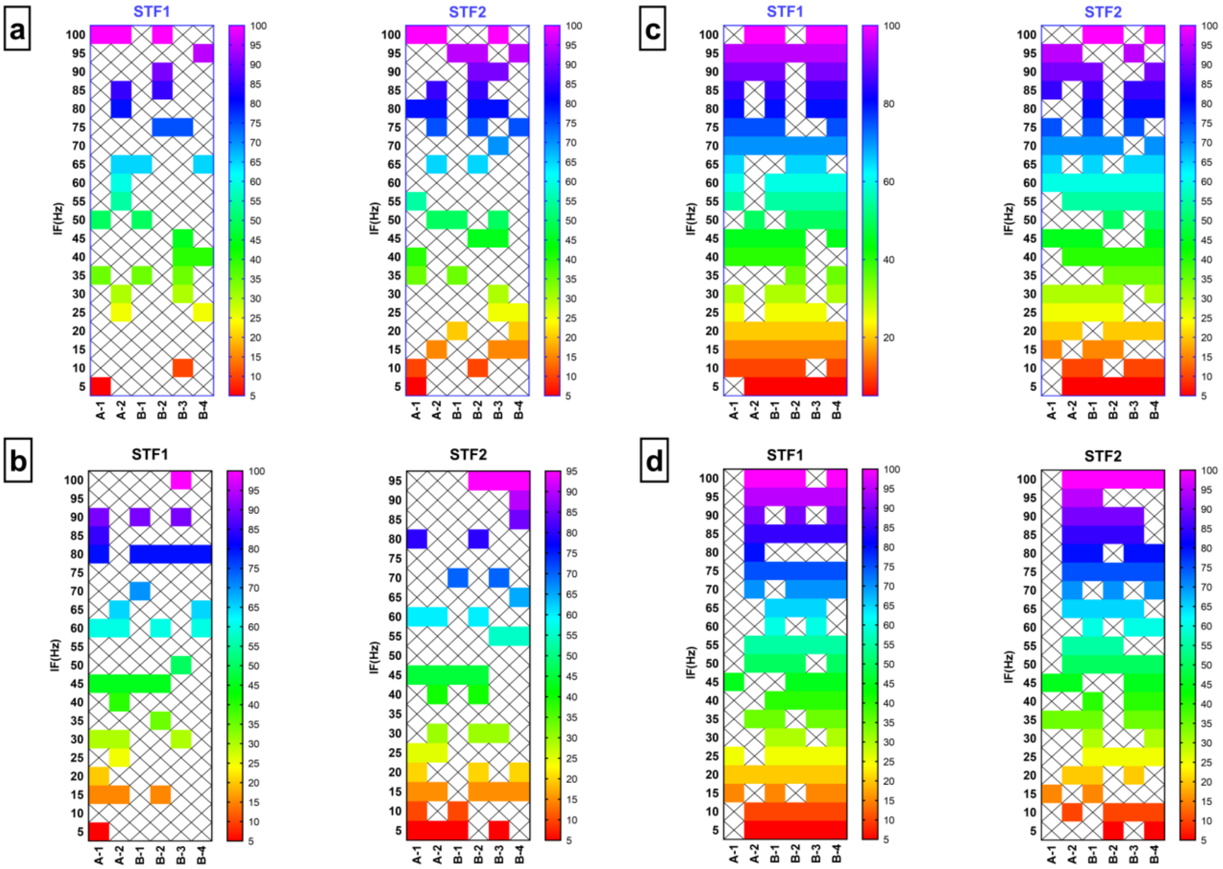

**Figure S.5**

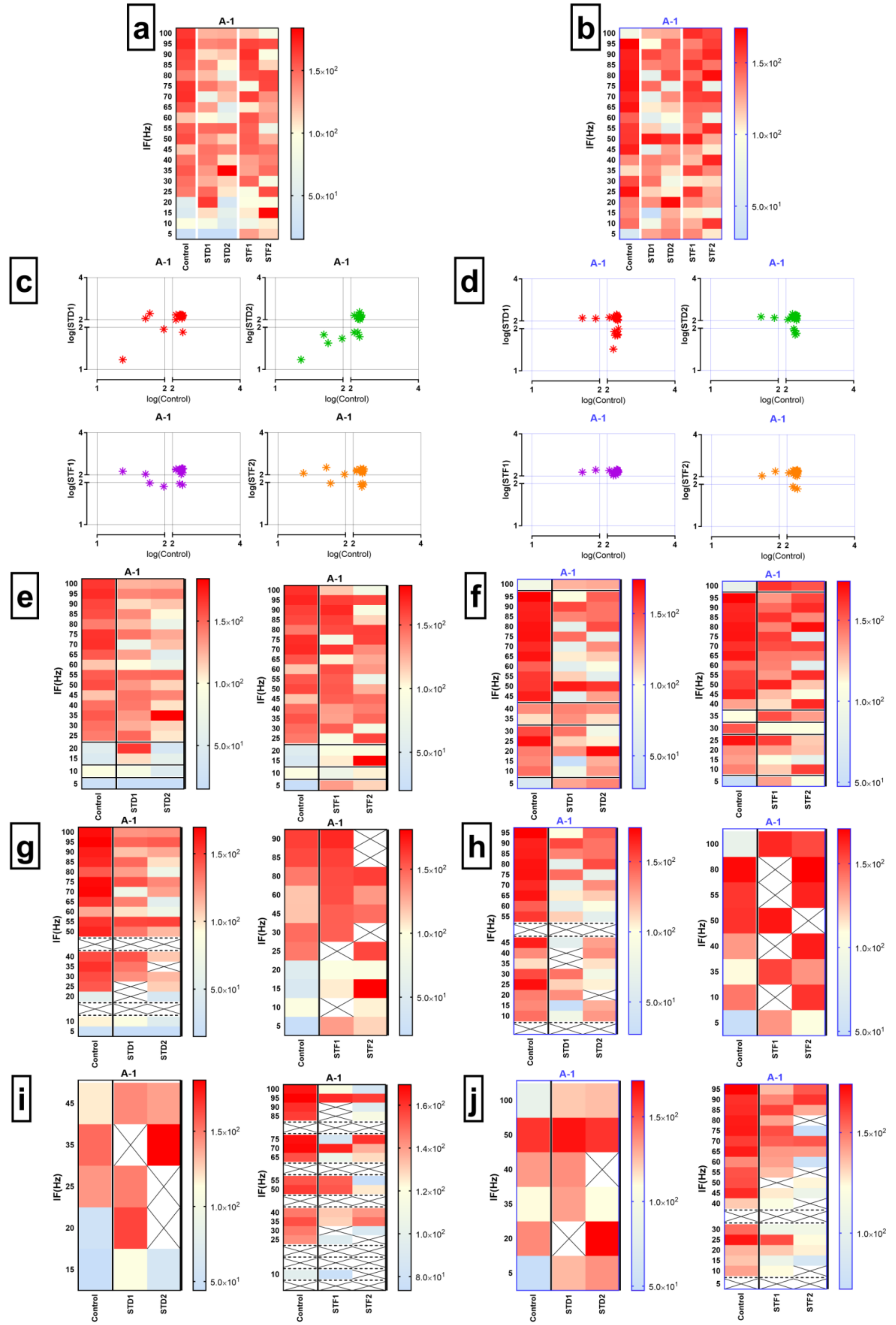
a
b
c
d
e
f
g
h
i
j
A-1
IF(Hz)
Control
STD1
STD2
STF1
STF2
log(Control)
log(STD1)
log(STD2)
log(STF1)
log(STF2)
1.5×10^2
1.0×10^2
5.0×10^1
1.6×10^2
1.4×10^2
1.2×10^2
8.0×10^1

**Figure S.6**

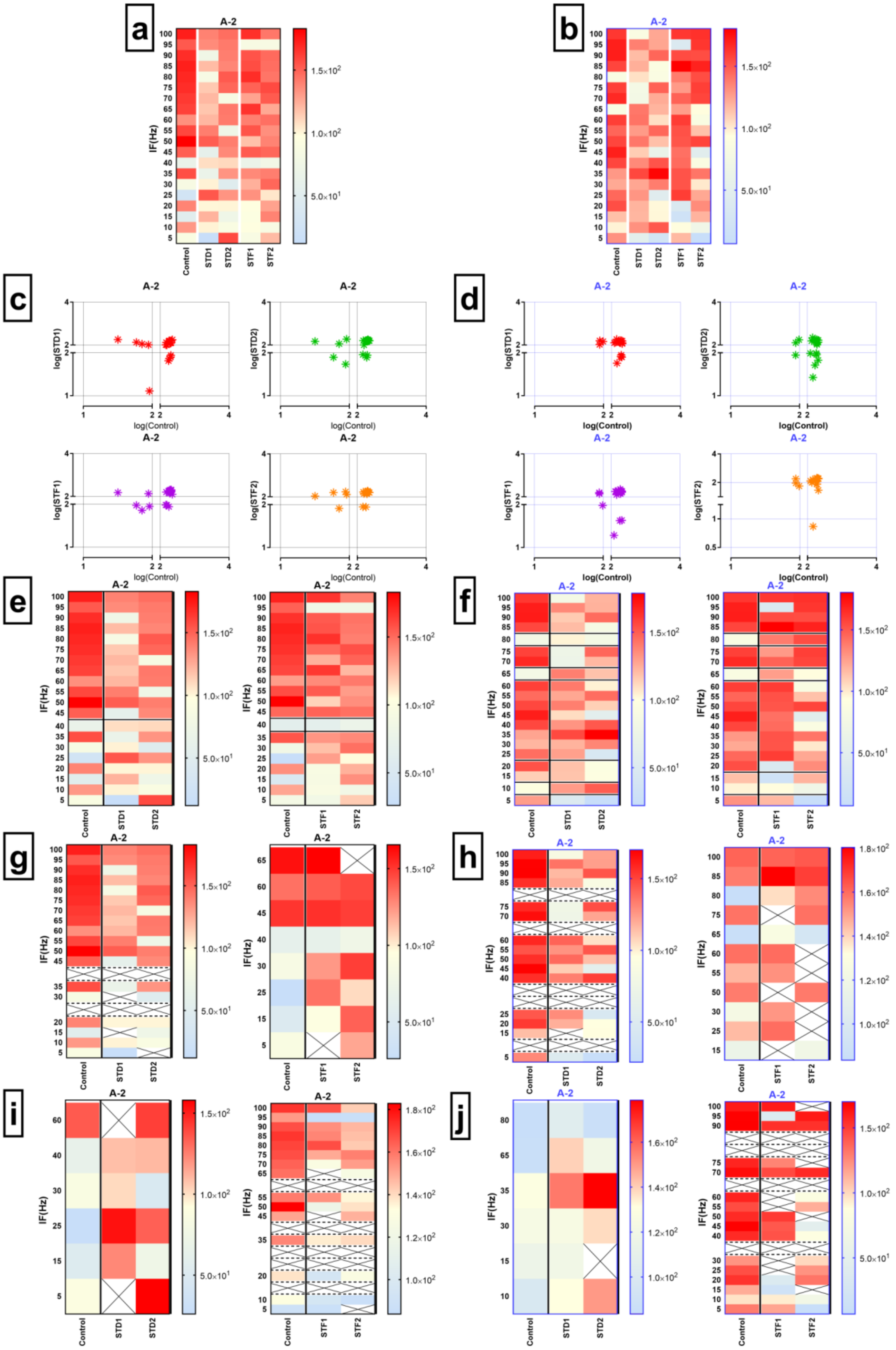
a
b
c
d
e
f
g
h
i
j
A-2
IF(Hz)
Control
STD1
STD2
STF1
STF2
log(Control)
log(STD1)
log(STD2)
log(STF1)
log(STF2)
1.5×10^2
1.0×10^2
5.0×10^1

**Figure S.7**

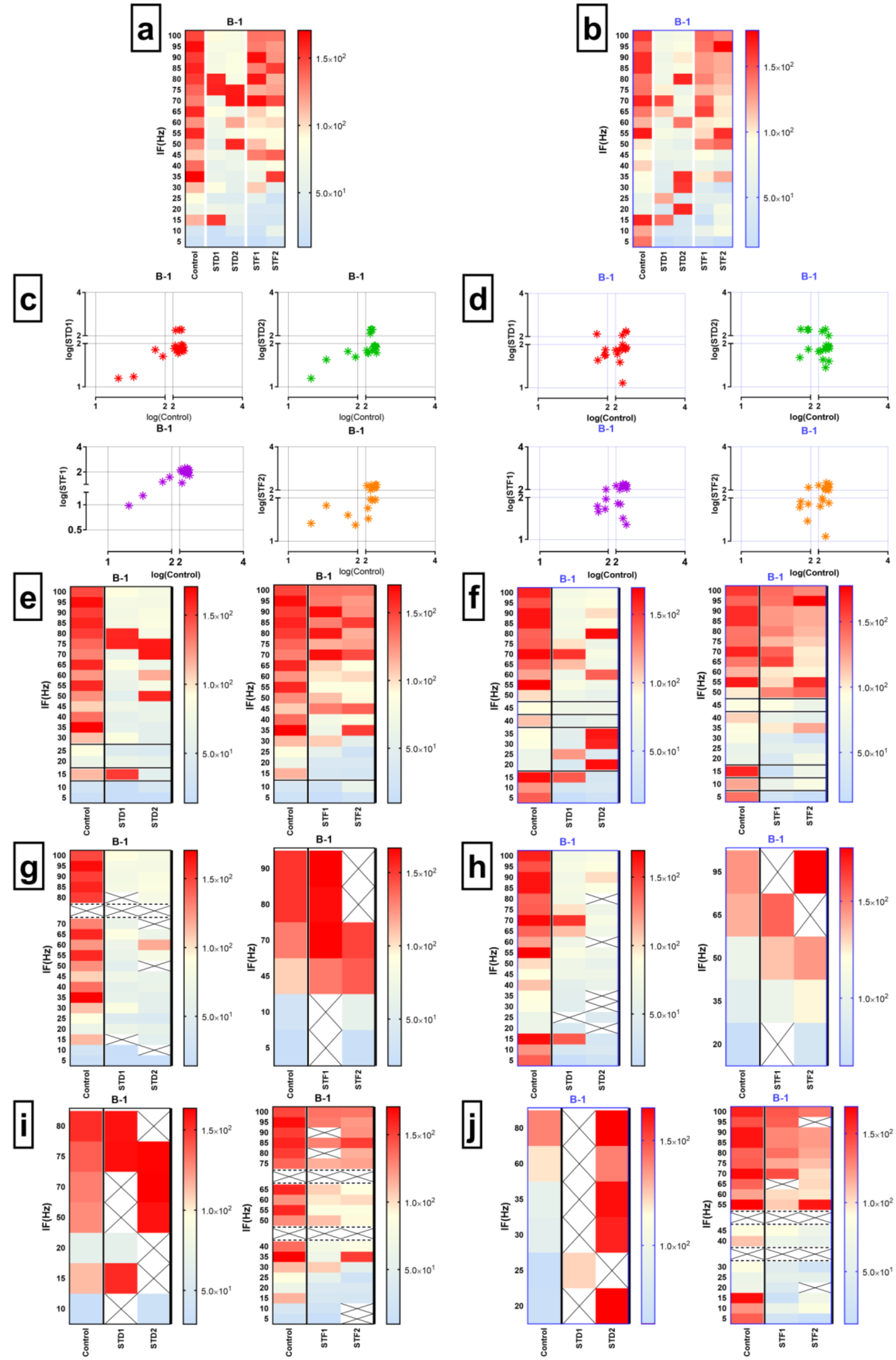
a
b
c
d
e
f
g
h
i
j
B-1
IF(Hz)
Control
STD1
STD2
STF1
STF2
log(Control)
log(STD1)
log(STD2)
log(STF1)
log(STF2)
1.5×10^2
1.0×10^2
5.0×10^1

**Figure S.8**

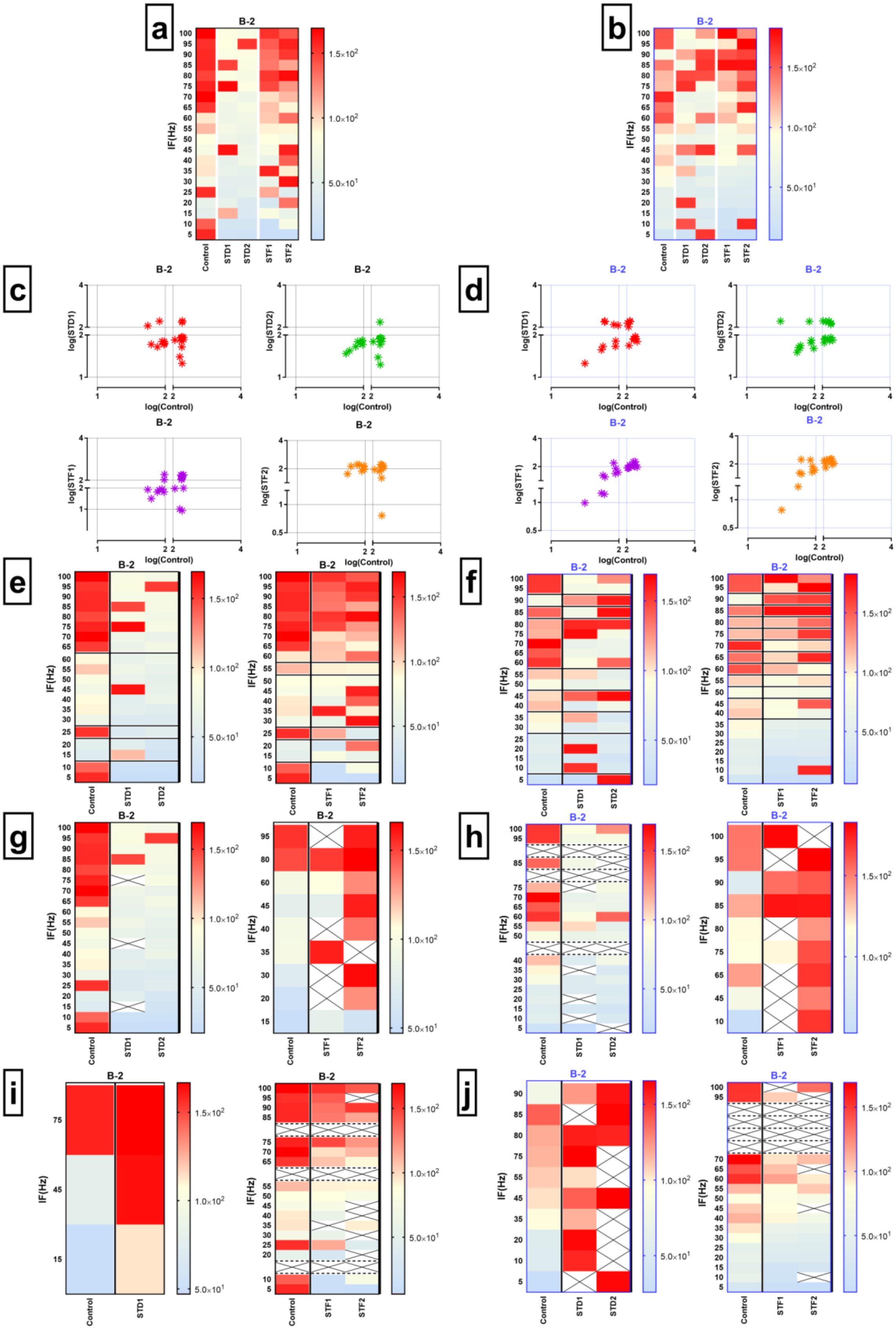
a
b
c
d
e
f
g
h
i
j
B-2
IF(Hz)
Control
STD1
STD2
STF1
STF2
log(Control)
log(STD1)
log(STD2)
log(STF1)
log(STF2)
1.5×10^2
1.0×10^2
5.0×10^1

**Figure S.9**

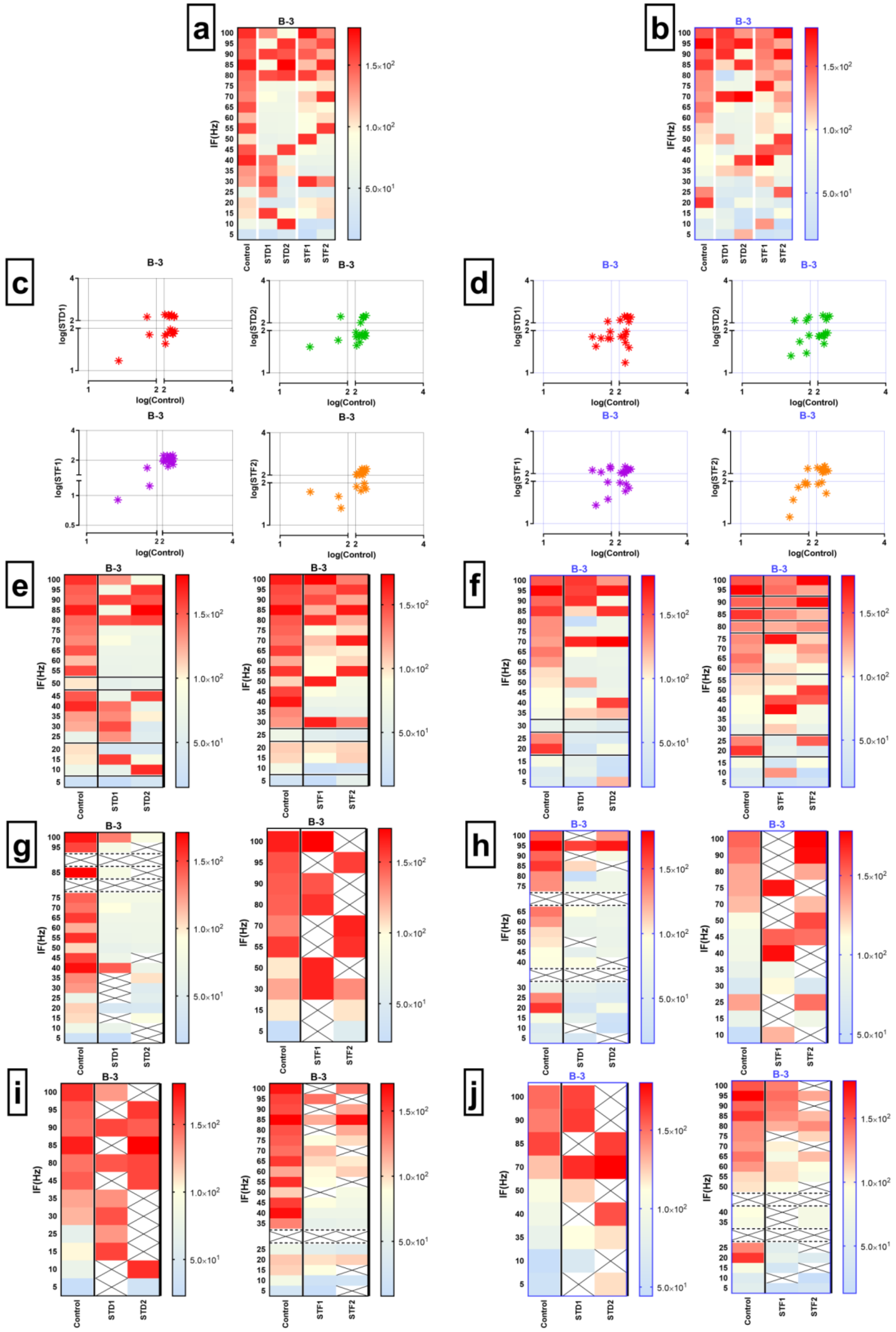
a
b
c
d
e
f
g
h
i
j
B-3
IF(Hz)
Control
STD1
STD2
STF1
STF2
log(Control)
log(STD1)
log(STD2)
log(STF1)
log(STF2)
1.5×10^2
1.0×10^2
5.0×10^1

**Figure S.10**

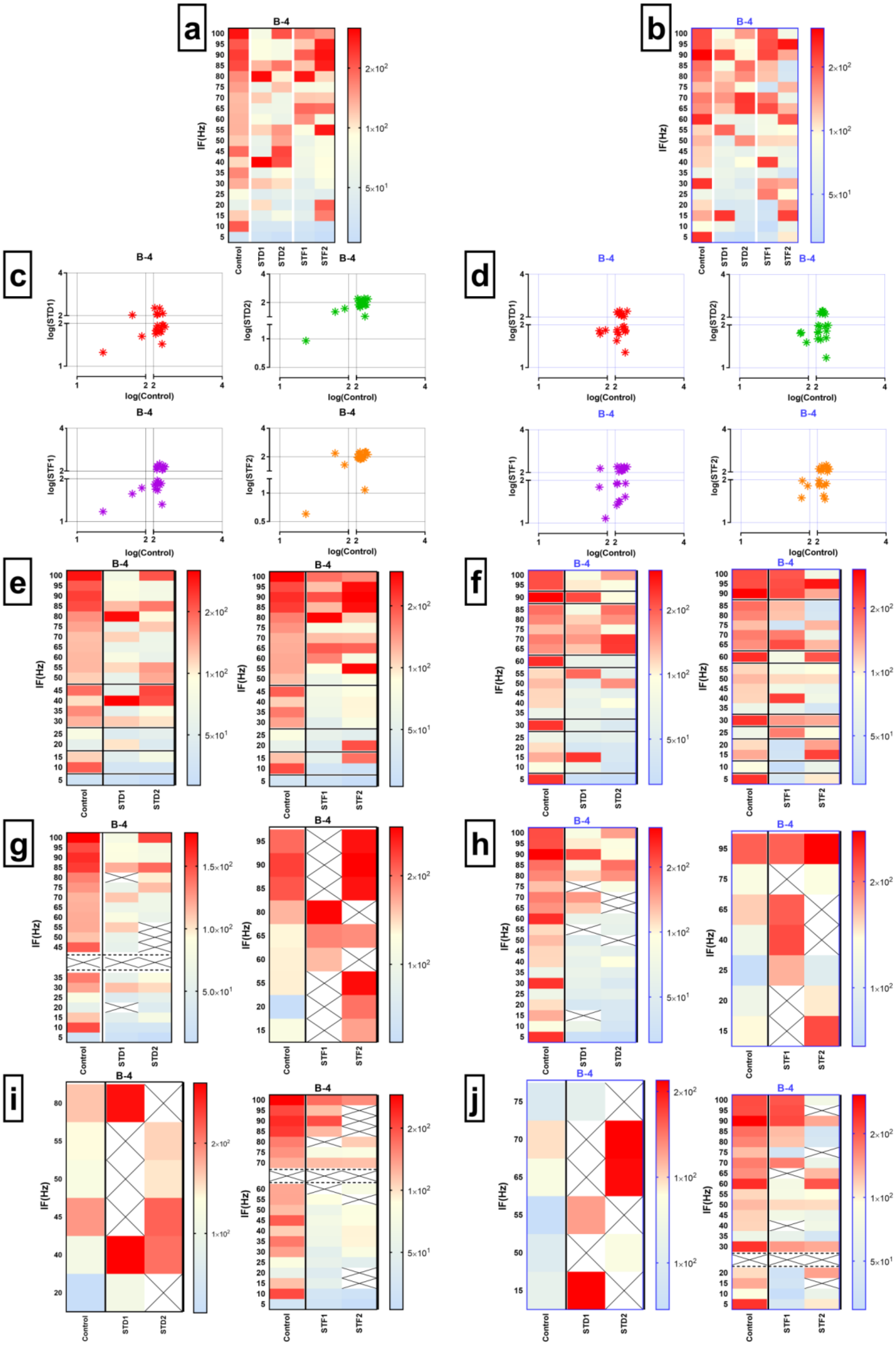
a
b
c
d
e
f
g
h
i
j
B-4
IF(Hz)
Control
STD1
STD2
STF1
STF2
log(Control)
log(STD1)
log(STD2)
log(STF1)
log(STF2)

## Supplementary Legends

**Figure S.1: Physiological responses of our network model at mV level at testing stage**. The graph refers to several connectivity conditions represented in seven panels namely: (a), (b1), (b2), (b3), (c1), (c2) and (c3). These panels refer to monitoring the network model activity in mV. A: shows connectivity between the following neuronal populations; (1) excitatory populations (E), (2) inhibitory populations (I), and (3) both neuronal populations (I and E). b1: refers to connectivity in (E). b2: indicates connectivity in (I). b3: represents connectivity in each neural population (I and E) separately. Panels (c1), (c2) and (c3) represent connectivity; between both neuronal populations (I and E), in each neuronal population, and a combination of (c1) & (c2) respectively.

**Figure S.2: Neural activity of b3 in Figure S.1.** Panels (A), (B), (C), (D) and (E) represent an ascending increase in the timing scale windows (seconds).

**Figure S.3:** Selected input frequency that succeeded in triggering modulation effect after implementing depressing dynamical synapses across all network scenarios of both conditions: immature *(panels (a) and (c))* and mature networks *(panels (b) and (d))*. *Panels (a) and (b)* refer to predicted modulated responses while *panels (c) and (d)* relate to unpredicted modulated responses.

**Figure S.4:** Selected input frequency that succeeded in triggering modulation effect after the implementation of facilitating dynamical synapses across all network scenarios of both conditions: immature *(panels (a) and (c))* and mature networks *(panels (b) and (d))*. *Panels (a) and (b)* refer to predicted modulated responses while *panels (c) and (d)* relate to unpredicted modulated responses.

**Figures; S.5, S.6, S.7, S.8, S.9 and S.10** represent the initial heat maps of the produced firing rate activity of the network scenarios; (A-1), (A-2), (B-1), (B-2), (B-3) and (B-4) respectively before and after implementing dynamical synapses; STD1, STD2, STF1 and STF2. The Y axes refer to the Poisson input frequencies (IF). *Panel (a)* refers to immature conditions while *panel (b)* relates to mature conditions. *Panel (c)* and *panel (d)* refers to the corresponding correlation before and after implementing STP, which relate to the immature and mature conditions, respectively. *Panels (e), (g)* and *(i)* refers to immature conditions while *panels (f), (h)* and *(j)* relates to mature conditions. *Panel (e)* and *panel (f)* refer to the overall modulated responses elicited by the depressing and facilitating synapses, respectively, for both network conditions. *Panel (g)* and *panel (h)* refer to the predicted modulated responses elicited by the depressing and facilitating synapses, respectively, for both network conditions. *Panel (i)* and *panel (j)* refer to the unpredicted modulated responses elicited by the depressing synapses and facilitating synapses, respectively, for both network conditions.
