# Supplementary material for: Dynamic Communications Between GABAA Switch, Local Connectivity, and Synapses During Cortical Development: A Computational Study
Source: Front Cell Neurosci. 2018 Dec 17;12:468. doi: 10.3389/fncel.2018.00468 (PMC6304749; doi:10.3389/fncel.2018.00468)
Supplement: Supplementary file 2 [file Data_Sheet_1.docx]

# A. Appendix

**A1. Model and Simulation Description**

**Table A1: Model Description, Simulation Paradigm and Parameters.** This tabular model description chosen was inspired from many studies *(Nordlie et al., 2009; Vogels et al., 2011; Lonardoni et al., 2017).*

# A1. Model and Simulation Description

| AMODEL SUMMARY | | | |
| --- | --- | --- | --- |
| Populations | | **Three: excitatory, inhibitory, external input** | |
| Connectivity | | **See C: CONNECTIVITY** | |
| Neuron Model | | **Conductance based leaky integrate and fire (LIF)** | |
| Synapse Model and Synaptic Dynamics | | | |
| Synapse Model | | **The synaptic interactions between these neurons were modeled as transient conductance changes. The synaptic time course was modeled as an instantaneous rise followed by an exponential decay.** $\frac{d\mathbf{g}_{\boldsymbol{exc}}}{dt}=-\mathbf{g}_{\boldsymbol{exc}}\boldsymbol{/}\boldsymbol{\tau}_{\boldsymbol{exc}}$ **_and_** $\left. \frac{d\mathbf{g}_{\boldsymbol{inh}}}{dt}=-\mathbf{g}_{\boldsymbol{inh}}\boldsymbol{/\tau}_{\boldsymbol{inh}} \right\}$ ***Equation 1.2*** | |
| Synaptic Dynamics | | $\left. \boldsymbol{\tau}_{\boldsymbol{syn}}\frac{\mathbf{d}\mathbf{g}_{\boldsymbol{syn}} \left( \mathbf{t} \right)}{\mathbf{dt}}\boldsymbol{= -}\mathbf{g}_{\boldsymbol{syn}}\left( \boldsymbol{t} \right)\boldsymbol{+}\mathbf{S}_{\boldsymbol{syn}}\boldsymbol{(t)} \right\}\boldsymbol{Equation 1.3}$  $\left. S_{syn}\left( t \right)=\sum_{i,k} ᵟ(t-t_{i}^{k}) \right\}\boldsymbol{Equation 1.4}$ | |
|  |  | Throughout equation 1.3, $\boldsymbol{\tau}_{\boldsymbol{syn}}$ refers the synaptic time constant while $\mathbf{g}_{\boldsymbol{syn}}$ represents the synaptic conductance, which is expressed in units of the leak conductance.  Equation 1.4 refers to the incoming synaptic spike trains where i belongs to {1,….., N}, which refers to presynaptic neurons and k indicates their different spike times of these neurons whereas *Syn ϵ {exc, inh}*. | |
| Spiking | | $If V\left( t-dt \right)< \theta\wedge V(t)\geq\theta$ 1-set t*=t  2-emit spike with time-stamp t* | |
| Spatial Organization | | **A cortical area of L = 1 mm^2^ was simulated as a 2D-layer-like network with periodic boundary conditions and an excitatory/inhibitory neuron number ratio of 4:1, respectively.** | |
| Input | | **Independent Poisson spike trains** | |
| Measurement | | **Firing Activity (Mean Spike Frequency, MSF)** | |
| B  POPULATIONS | | | |
| Name | **Elements** | | **Size** |
| E | LIF neuron | | n_exc = 4 n_inh |
| I | LIF neuron | | n_inh |
| Ex | Excitatory Poisson input generator | | one realization per neuron |

| C  CONNECTIVITY | | | |
| --- | --- | --- | --- |
| C.1:Name | Source | Target | Pattern |
| EE | E | E | C_EE_=connect all excitatory neurons to all excitatory neurons within a given distance x, weight J, delay d |
| IE | I | E | C_IE_=connect all inhibitory neurons to all excitatory neurons within a given distance x, weight J, delay d |
| EI | E | I | C_EI_=connect to all excitatory neurons to all inhibitory neurons within a given distance x, weight −gJ, delay d |
| II | I | I | C_II_=connect to all inhibitory neurons to all inhibitory neurons within a given distance x, weight −gJ, delay d |
| Ex | Ex | E ∪ I | C_EX_=independent Poisson spike trains, weight Jx, rate νEx |
| C.2:Network Scenarios  see also figure1 of Khalil et al., 2017 | Percentages of local density of dendritic arborization (ϵ) | Percentages of lateral spread length between neighboring neurons (Local connectivity) (δ) | |
| (A-1) | 1% | 9% | |
| (A-2) |  | 10% | |
| (B-1) | 9% | 1% | |
| (B-2) | 10% |  |  |
| (B-3) | 19% |  |  |
| (B-4) | 20% |  |  |
| Equations | | | |
| $\left. \boldsymbol{p}_{\boldsymbol{ij}}\boldsymbol{=}\boldsymbol{e}^{\boldsymbol{-}\frac{\boldsymbol{r}_{\boldsymbol{ij}}^{\boldsymbol{2}}}{\boldsymbol{2}\boldsymbol{\delta}_{\boldsymbol{c}}^{\boldsymbol{2}}}} \right\}\boldsymbol{Equation 2.1}$ | | | |
| $\left. \boldsymbol{N}_{\boldsymbol{realized}}\left( \boldsymbol{r} \right)\boldsymbol{=}\mathbf{NP}\left( \mathbf{r} \right)\mathbf{exp(-}\boldsymbol{r}^{\boldsymbol{2}}\mathbf{/2}\boldsymbol{\sigma}_{\boldsymbol{c}}^{\boldsymbol{2}}\mathbf{)} \right\}\boldsymbol{Equation 2.2}$  $\left. \boldsymbol{\rho}\left( \boldsymbol{r} \right)\boldsymbol{=}\mathbf{P}\left( \mathbf{r} \right)\mathbf{ex}\mathbf{p} \left( \mathbf{-}\frac{\boldsymbol{r}^{\boldsymbol{2}}}{\mathbf{2}\boldsymbol{\sigma}_{\boldsymbol{c}}^{\boldsymbol{2}}} \right) \right\}\boldsymbol{Equation 2.3}$ | | | |
| $\left. \mathbf{d}_{\boldsymbol{ij}}\boldsymbol{=}\mathbf{d}_{\boldsymbol{syn}}\boldsymbol{+}\frac{\boldsymbol{rij}}{\boldsymbol{v}} \right\}\boldsymbol{Equation 2.4}$ | | | |
| For further details, see Gaussian-distributed local connectivity profile and Non-homogenous propagation delay | | | |

| D  INPUT | |
| --- | --- |
| Type | Description |
| Poisson Generators | Rate νEx, projects independent realizations to all neurons |

| F  SHORT-TERM SYNAPTIC PLASTICITY | |
| --- | --- |
| Parameter | **Description** |
| STD | U= 0.6. Large U means that an initial spike incurs a large drop in the fraction of resources that remain available after neurotransmitter depletion that takes a long time to recover. |
| STD1 | Synaptic Time Constant for depression (*τd*) equal to **100** milliseconds while Synaptic Time Constant for facilitation (*τf*) equal to **1** millisecond. |
| STD2 | Synaptic Time Constant for depression (*τd*) equal to **100** milliseconds while Synaptic Time Constant for facilitation (*τf*) equal to **10** milliseconds. |
| STF | U= 0.1. Small *U* means that synaptic efficacy is gradually increased by spikes. |
| STF1 | Synaptic Time Constant for facilitation (τf) equal to 100 milliseconds while Synaptic Time Constant for depression (*τd*) equal to **1** milliseconds. |
| STF2 | Synaptic Time Constant for facilitation (τf) equal to 100 milliseconds while Synaptic Time Constant for depression (*τd*) equal to **10** milliseconds. |
| STP influences Ex-connectivity(i.e., connectivity based on external Poisson input, see C:CONNECTIVITY) | |
| Phenomenological model Description | |
| $\frac{\boldsymbol{du}}{\boldsymbol{dt}}\boldsymbol{= -}\frac{\boldsymbol{u}}{\boldsymbol{\tau}{}_{\boldsymbol{f}}}\boldsymbol{+U}\left( \boldsymbol{1-}\boldsymbol{u}^{\boldsymbol{-}} \right)\boldsymbol{\delta}\left( \boldsymbol{t-}\boldsymbol{t}_{\boldsymbol{sp}} \right)\boldsymbol{,}$  $\frac{\boldsymbol{dx}}{\boldsymbol{dt}}\boldsymbol{=}\frac{\boldsymbol{1-x}}{\boldsymbol{\tau}{}_{\boldsymbol{d}}}\boldsymbol{-}\boldsymbol{u}^{\boldsymbol{+}}\boldsymbol{x}^{\boldsymbol{-}}\boldsymbol{\delta}\left( \boldsymbol{t-}\boldsymbol{t}_{\boldsymbol{sp}} \right)\boldsymbol{,}$  “The joint effect of ux is determined by being dominated either by depression or by facilitation. In the parameter τd≫τf and substantial U, a starting spike acquires a large drop in x that takes a long time to recover. However, when τf≫τd and the U value is small then, synaptic efficacy is gradually increased by spikes. Consequently, dynamical synapses become facilitating synapses, STF”. (Khalil et al, 2017 a; b) | |

Table A1: Model Description, Simulation Paradigm and Parameters.

| **E**  **MODEL PARAMETERS** | | | |
| --- | --- | --- | --- |
| **Biophysical Parameters** | **Symbol** | **Value** | **References** |
| **Neurons** | | | |
| **Number of Neurons** | **N** | 3000 | See Appendix B: Benchmark Simulation of Brette *et al.*, (2007) |
| **Number of Excitatory neurons** | **n__exc_** | 2400 |  |
| **Number of Inhibitory Neurons** | **n__inh_** | 600 |  |
| **Size of Network** | **Size** | 1mm^2^ | (Yger *et al.*, 2011) |
| **Membrane Parameters** | | | |
| **Membrane Capacitance** | **c_m** | 0.2 nF | (Yger *et al.*, 2011) |
| **Membrane Time Constant** | ***τ*_m_** | 20 ms | (Song *et al.*, 2000; Brette *et al.*, 2007; Vogels *et al.*, 2011; Yger *et al.*, 2011) |
| **Glutamatergic Synaptic Time Constant for AMPA** | ***τ*_exc_** | 5 ms | (Brunel, 2000; Song *et al.*, 2000; Song & Abbott, 2001; Vogels *et al.*, 2011)  (Brunel, 2000; Vogels *et al.*, 2011; Yger *et al.*, 2011) |
| **GABAergic Synaptic Time Constant for GABA_A_** | ***τ*_inh_** | 10 ms |  |
| **Refractory Time Constant(Absolute refractory period)** | ***τ*_ref_** | 5 ms | (Brette *et al.*, 2007; Vogels *et al.*, 2011; Yger et al., 2011) |
| **Synaptic delay** | **d_syn_** | 0.2 ms | (Yger *et al.*, 2011) |
| **Threshold Potential and Reversal Potential** | | | |
| **Leak Reversal Potential** | **El** | -70.6 mV | (Brette & Gerstner, 2005) |
| **Excitatory Reversal Potential for AMPA** | **Ee** | 0 mV | (Brunel, 2000; Song *et al.*, 2000; Song & Abbott, 2001; Brette & Gerstner, 2005; Clopath *et al.*, 2010; Yger *et al.*, 2011) |
| **Resting Membrane Potential** | **V_rest_** | -74 mV | (Song & Abbott, 2001 ) |
| **GABA_A’_ Reversal Potential for Immature Neocortical Network** | **E_GABA_** | -40mV | *(Rheims et al., 2008)* |
| **GABA_A’_ Reversal Potential for Mature Neocortical Network** | **Ei** | -70 mV | (Song *et al.*, 2000; Vogels *et al.*, 2011; Yger *et al.*, 2011) |
| **Threshold Potential (Spiking threshold)** | **V_t_** | -50.4 mV | (Brette & Gerstner, 2005) |
| **Conductance** | | | |
| **Leak Conductance** | **G_Leak_** | 10 nS | (Vogels *et al.*, 2011;Yger *et al.*, 2011) |
| **Decay constant of AMPA-type conductance(Excitatory Gluatamergic Conductance (AMPA))** | **g_exc_** | 4 nS | (Yger *et al.*, 2011) |
| **Decay constant of GABA-type conductance(Inhibitory GABAergic Conductance (GABA_A_))** | **g_inh_** | 64 nS | (Yger *et al.*, 2011) |
| **Conductance for The External Input Drive (Poisson Input)** | **g_ext_** | 200 nS | (Yger *et al.*, 2011) |
| **Conductance Velocity**  **(The transmission delay depends on the Euclidean distance between neurons)** | **C_vol_** | 0.5 mm/ms | (Bringuier *et al.*, 1999; González-Burgos *et al.*, 2000) |
| **Poisson Input Frequency(IF)** | | | |
| **Ranges of Poisson Input Frequency(IF)** | **ν_Ex_** | 5 to 100 Hz with 5 Hz interval, i.e., 5Hz, 10Hz, 15 Hz…ect | |
